# Trajectories and dimensional phenotypes of depressive symptoms throughout pregnancy and postpartum in relation to prior premenstrual symptoms

**DOI:** 10.1192/bjp.2025.38

**Published:** 2025-06

**Authors:** Ella Schleimann-Jensen, Inger Sundström-Poromaa, Samantha Meltzer-Brody, Tory A. Eisenlohr-Moul, Fotis C. Papadopoulos, Alkistis Skalkidou, Erika Comasco

**Affiliations:** Department of Women’s and Children’s Health, Science for Life Laboratory, Uppsala University, Sweden; Department of Women’s and Children’s Health, Uppsala University, Sweden; Department of Psychiatry, University of North Carolina School of Medicine, USA; Department of Psychiatry, University of Illinois at Chicago, USA; Department of Medical Sciences, Uppsala University, Sweden

**Keywords:** Reproductive psychiatry, women, ovarian hormones, premenstrual disorder, perinatal depression

## Abstract

**Background:**

Sensitivity to ovarian hormone fluctuations can lead to mental distress during the luteal phase of the menstrual cycle, such as in premenstrual syndrome (PMS) and premenstrual dysphoric disorder (PMDD), and also during pregnancy and postpartum, as in perinatal depression (PND).

**Aims:**

In two cohorts, we investigated the relationship between history of PMS/PMDD and PND symptoms. We also examined how premenstrual symptoms are associated with perinatal symptom trajectories and dimensional phenotypes of PND symptoms, which remains unidentified.

**Method:**

From early pregnancy until 6 months postpartum, participants of two large longitudinal cohorts were followed using the Edinburgh Postnatal Depression Scale (EPDS). Premenstrual symptoms were self-reported retrospectively.

**Results:**

Both pre-pregnancy PMS and PMDD were associated with higher EPDS scores across pregnancy and postpartum, even after adjustment for confounders. The odds of developing PND were higher among those reporting PMS and PMDD, ranging up to 1.68 (95% CI 1.25–2.29) (6–13 weeks postpartum) and 3.05 (95% CI 2.26–4.10) (late pregnancy) respectively for PMS and PMDD, throughout the perinatal period. Premenstrual symptomatology was associated more with certain PND trajectories based on the time of occurrence and persistence of symptoms. However, PND symptom severity did not differ depending on premenstrual symptomatology in any trajectory. Prior PMS/PMDD was associated with underlying dimensions of symptom constructs of PND, including severe and moderate symptoms of depressed mood, anxiety and anhedonia.

**Conclusions:**

Women with a history of PMS/PMDD require coordinated care by psychiatrists, other mental health clinicians, midwives and gynaecologists during pregnancy as well as postpartum.

Women are more prone to develop mental distress concomitant with ovarian hormone fluctuations associated with reproductive events. Ovarian hormone sensitivity throughout the reproductive lifespan may therefore be a risk factor for mood and affective disorders.

## Premenstrual syndrome (PMS) and premenstrual dysphoric disorder (PMDD)

Premenstrual dysphoric disorder (PMDD) is a hormone-related mood disorder characterised by cyclic affective, cognitive and physical symptoms peaking in the late luteal phase of the menstrual cycle and subsiding in the follicular phase.^1^ Core symptoms of PMDD include depression, anxiety, affective lability and irritability, which result in an individual and societal burden comparable to major depression and affecting about 2–5% of women.^1,2^ Furthermore, nearly half of those with PMDD experience suicidal ideation.^3^ However, PMDD represents only the tip of the iceberg, as 20–30% of those who menstruate report premenstrual syndrome (PMS) – a less severe form of the same symptoms.^4,5^

Although having a significant impact throughout a large proportion of the individual’s life, PMS and PMDD may also serve as risk factors for mental ill health during the peripartum period.^5^ The same sensitivity to hormones observed across cycles^6^ may also manifest as symptoms during pregnancy and postpartum, termed perinatal depression (PND).^7^

## Perinatal depression (PND)

Perinatal depression affects about 12–25% of those giving birth^8,9^ and can have serious implications for the child’s development and health.^10^ The onset of PND symptoms occurs during pregnancy or within 4 weeks after delivery, according to DSM-5, and symptoms include: depression, anxiety, fatigue, loss of interest in activities, concentration difficulties, sleep and appetite disturbances, feelings of being a bad mother or parent and a fear of harming the baby or oneself. Beyond the personal and societal burden of the disease,^11^ guilt and suicidal thoughts are also common.^12^ Suicide accounts for about 20% of maternal deaths.^13,14^

Although triggered by the hormonal changes of pregnancy, the pathogenesis of PND is likely multifactorial, with variation in multiple physiological systems being relevant.^15^ In addition, different trajectories of the disorder may exist, with varying onset and persistence of symptoms in relation to sensitivity to reproductive hormones.^16–18^ Furthermore, dimensional phenotypes of PND symptoms have been differentially associated with severity of depression, anxiety, suicidal ideation and symptom onset.^19^ Based on the three phenotypic dimensions of depressed mood, anxiety and anhedonia, one study identified five subtypes of PND: severe anxious depression, moderate depression with anxiety, anxious anhedonia, pure anhedonia and resolved depression.^20^

## Knowledge gap and aims

To date, evidence for an association between PND and PMS/PMDD remains inconsistent, recently deemed unconvincing and mainly based on studies with serious risk of bias.^21^ Indeed, the existing mix of study designs, vague definitions of PMS or PMDD (i.e. most studies included only PMS) and small sample sizes undermine our understanding of the relationship between PMS/PMDD and PND. Moreover, most studies focused only on the postpartum period and considered PMS and/or PMDD as covariates. Thus, longitudinal assessments of PND are also lacking, and the temporal dynamics and phenotypic dimensions of PND in relation to symptoms of PMS/PMDD remain uninvestigated.

Currently, patients suffer owing to a lack of coordinated care among psychiatrists, mental health clinicians and gynaecologists to address how reproductive hormone sensitivity may unfold across multiple reproductive transitions in a single patient. This gap underscores the urgent need for a better understanding of how reproductive events such as pregnancy and the menstrual cycle affect mental health in shared or unique ways. The present study thus aimed to address whether prior premenstrual symptomatology is a risk factor for PND at any time point during both pregnancy and postpartum, accounting for psychosocial factors. It also sought to investigate how history of PMS/PMDD relates to trajectories of onset and persistence of PND symptoms, as well as dimensional constructs of symptoms of PND. In this study, these questions were addressed in two large, prospective cohorts who were followed throughout pregnancy and 6 months postpartum.

## Method

### Study design and population

In this observational prospective cohort study, participants from the Biology, Affect, Stress, Imaging and Cognition (BASIC; www.basicstudie.se) (*n* = 4569)^22^ and Mom2B (www.mom2b.se) (*n* = 4591)^23^ studies, two large population-based cohorts of women assessed from early or mid-pregnancy up to 1 year postpartum, were considered (Supplementary Material, available at https://doi.org/10.1192/bjp.2025.38). As the present study focuses on outcomes related to reproductive health, it is inherently sex-specific and includes only female participants, regardless of their gender identity. When using the term women throughout the text, we refer to those who were assigned the female sex at birth. The Uppsala Regional Ethical Committee approved the BASIC study (Dnr 2009/171) and the Swedish Ethical Review Authority approved the Mom2B study (Dnr 2019-01170); both were conducted according to the principles of the Helsinki Declaration of 1975, as revised in 2013. All participants gave written informed consent.

### Procedures

**Fig. 1:**
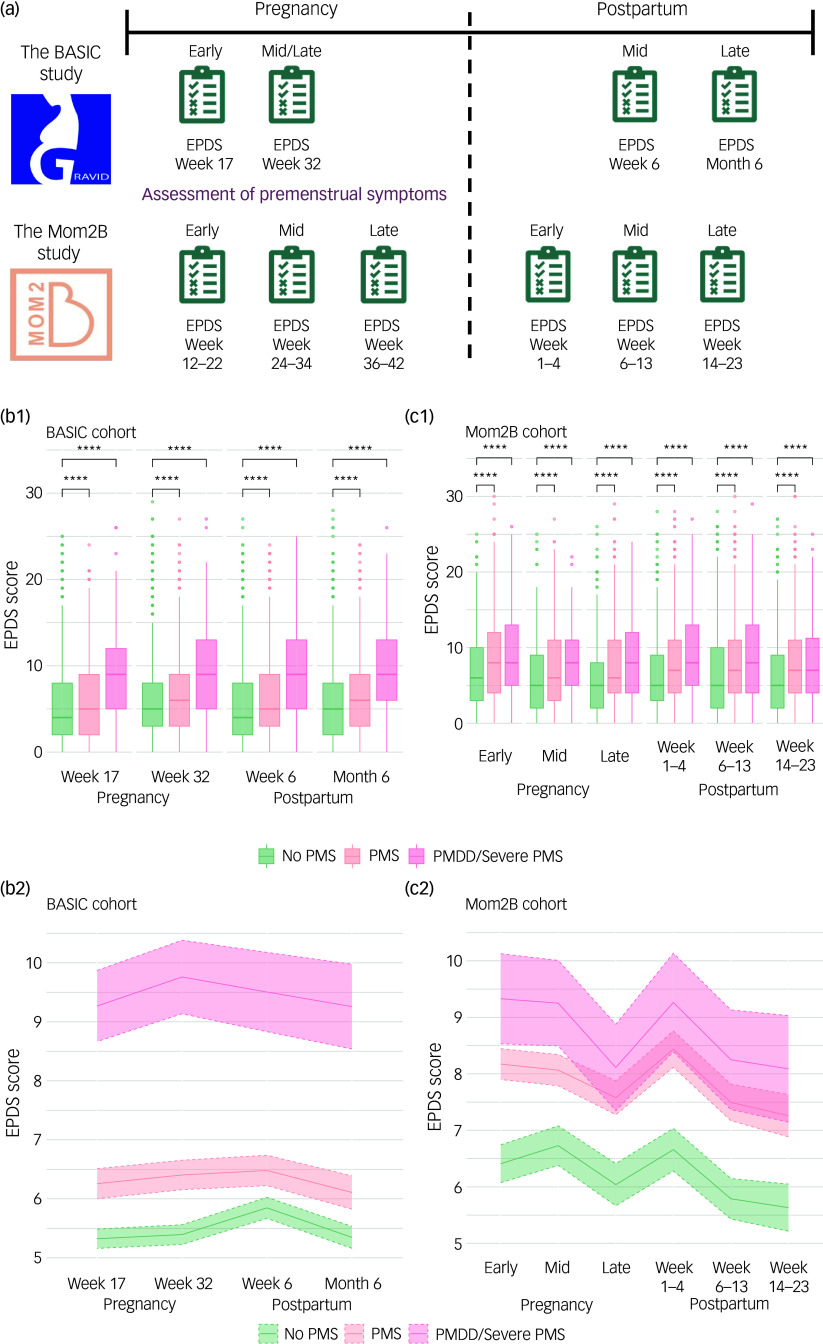
Study design and perinatal depression (PND) symptom severity in relation to premenstrual symptomatology over time. (a) The study design of the BASIC and Mom2B studies showing the time points for completion of the Edinburgh Postnatal Depression Scale (EPDS); (b1) and (c1) group differences on continuous EPDS scores at all time points during pregnancy and postpartum between those with no premenstrual syndrome (No PMS), with premenstrual syndrome (PMS) and with premenstrual dysphoric disorder (PMDD) (BASIC)/severe PMS (Mom2B) for the BASIC and Mom2B cohorts respectively; (b2) and (c2) mean EPDS scores over all time points during pregnancy and postpartum by group: no PMS, PMS and PMDD (BASIC)/severe PMS (Mom2B) for the BASIC and Mom2B cohorts respectively. Shaded areas indicate 95% confidence intervals. Statistical significance was set at *P* < 0.05. ****P ≤ 0.0001.

The Edinburgh Postnatal Depression Scale (EPDS)^24–26^ was administered to all participants. In the BASIC cohort, pregnant participants in Uppsala completed the EPDS at two time points during pregnancy and at two time points postpartum, whereas in the Mom2B cohort the EPDS was completed at three time points during pregnancy and at three time points postpartum. For convenience we will refer to these as early (week 17)/mid/late (week 32) pregnancy and early (week 6)/late (month 6) postpartum for the BASIC cohort, and as early (week 12–22)/mid (week 24–34)/late (week 36–42) pregnancy and early (week 1–4)/mid (week 6–13)/late (week 14–23) postpartum for the Mom2B cohort (Fig. 1(a)). The total EPDS score was computed separately for each time point by summing the ten items, each rated on a scale from 0 to 3. The participants were categorised into PND cases versus controls for each time point, using a EPDS cut-off score of ≥12 to indicate clinically relevant depressive symptoms.^25,27^ The participants were further assigned to four severity categories (no depression, mild to moderate, moderate to severe and very severe^20^) at each time point, and into trajectory groups based on onset and persistence of symptoms (controls, gestational PND, postpartum PND and persistent PND). Inspired by previous literature, dimensional constructs across time points were computed based on clustering of the ten EPDS items^20^ (cut-offs, time points and group definition respectively are given in the Supplementary Material).

In the BASIC cohort, premenstrual symptoms were retrospectively self-reported at gestational week 17. To be categorised as having PMDD, the participants had to have reported experiencing at least five symptoms mentioned in the DSM-5 criteria A–C for PMDD (Supplementary Material) before the onset of menses each month, out of which at least one had to be a core symptom. Additionally, in line with the DSM-5 criteria D, the symptoms had to interfere significantly with their daily life. PMS was defined using the ICD-10 criteria, which required that the participants had reported experiencing at least one of the symptoms asked about premenstrual symptoms in each menstrual cycle (Supplementary Material). In the Mom2B cohort, the participants reported at some point during pregnancy whether they had ever experienced PMS (yes/no) and whether they had ever been treated for severe PMS (yes/no). In the analyses, these were defined as ‘PMS’ and ‘severe PMS’ respectively.

### Covariates

Psychosocial data were collected through self-report at different time points during pregnancy or postpartum, with participants reporting on sociodemographic, gynaecological and obstetric information, lifestyle, sleep, psychological characteristics, antidepressant use and other treatment before, during or after pregnancy (Supplementary Material).

### Statistical analyses

All statistical analyses were performed in RStudio using R version 4.3.0 for Windows. Statistical significance was set at *P* < 0.05. Data concerning premenstrual symptomatology and PND status were cross-tabulated, as well as with possible confounders. Chi-squared tests were used for categorical variables and independent *t*-tests for continuous variables. A correlation matrix described multicollinearity between covariates. Among highly correlated variables, only one was retained in the models.

Regarding qualitative and quantitative associations between PND and premenstrual symptomatology, repeated measures analysis of variance (ANOVA) compared continuous EPDS scores across time points in those reporting no PMS versus PMS/PMDD (BASIC)/severe PMS (Mom2B). Pairwise comparisons with Bonferroni correction were applied on significant ANOVA results. Additionally, categories of PPD severity^20^ were investigated in relation to PMS/PMDD (BASIC)/severe PMS (Mom2B) using chi-squared tests.

Complementary multivariate logistic regression models were fitted separately for each time point, to investigate the odds of having PND as a function of premenstrual symptomatology, using the generalised linear model (GLM) framework with the binomial family and log link. To examine within-subject effects over the whole perinatal period, multivariable generalised estimating equation (GEE) models were additionally fitted, with binomial family, log link and independent correlation structure, clustered by participant. All models used PMS or PMDD (BASIC)/severe PMS (Mom2B) as the main exposure and a dichotomised EPDS score (threshold ≥12) as the outcome, and were adjusted for age, parity, history of depression and anxiety, antidepressant use before or during pregnancy, pregnancy complications and partner support.

Concerning PND symptom onset trajectories, multinomial logistic regression tested whether those reporting no PMS, PMS or PMDD (BASIC)/severe PMS (Mom2B) were more likely to develop a PND trajectory (although the sample size for the persistent PND trajectory was small owing to missing data) compared with the control trajectory. The impact of reports of no PMS/PMS/PMDD (BASIC)/severe PMS (Mom2B) on the EPDS scores for each PND trajectory based on symptom onset and persistence was investigated using the Kruskal–Wallis non-parametric test, followed by Dunn’s test for multiple comparisons for significant results.

Regarding the analysis of dimensional phenotypes of PND symptoms, a factor analysis explored underlying factors in the EPDS questions based on all observations over time, using scaled variables of the ten EPDS items from all participants, and this identified three factors with an eigenvalue above 1 (Supplementary Material). Inspired by Putnam et al,^20^ the research domain criteria (RDoC) functional constructs of negative valence and arousal were applied to the three dimensions of the EPDS: depressed mood, anxiety and anhedonia (Supplementary Material). PND symptoms clusters based on factor scores were found using *k*-means clustering, using *k* = 6 (Supplementary Material). The proportions of the identified PND symptoms clusters were then investigated grouped into no PMS, PMS and PMDD (BASIC)/severe PMS (Mom2B). Clusters were compared over time and between groups using chi-squared tests for single time points and McNemar’s test between different time points.

## Results

### Sample characteristics

Demographic and psychosocial characteristics of the participants are presented in Supplementary Tables S1 and S2 for the BASIC and Mom2B cohorts respectively, and the time points are shown in Fig. 1(a) and further described in the Supplementary Material. Regarding premenstrual symptoms, in the BASIC cohort, 30% reported PMS and 6% PMDD. In the Mom2B cohort, 55% reported PMS and 9% severe PMS. Mean EPDS scores differed between time points in both cohorts (Tables S1 and S2) and the proportion of PND cases consistently peaked around mid-/late pregnancy and early postpartum, and declined in the later postpartum time points (Table S3).

**Fig. 2:**
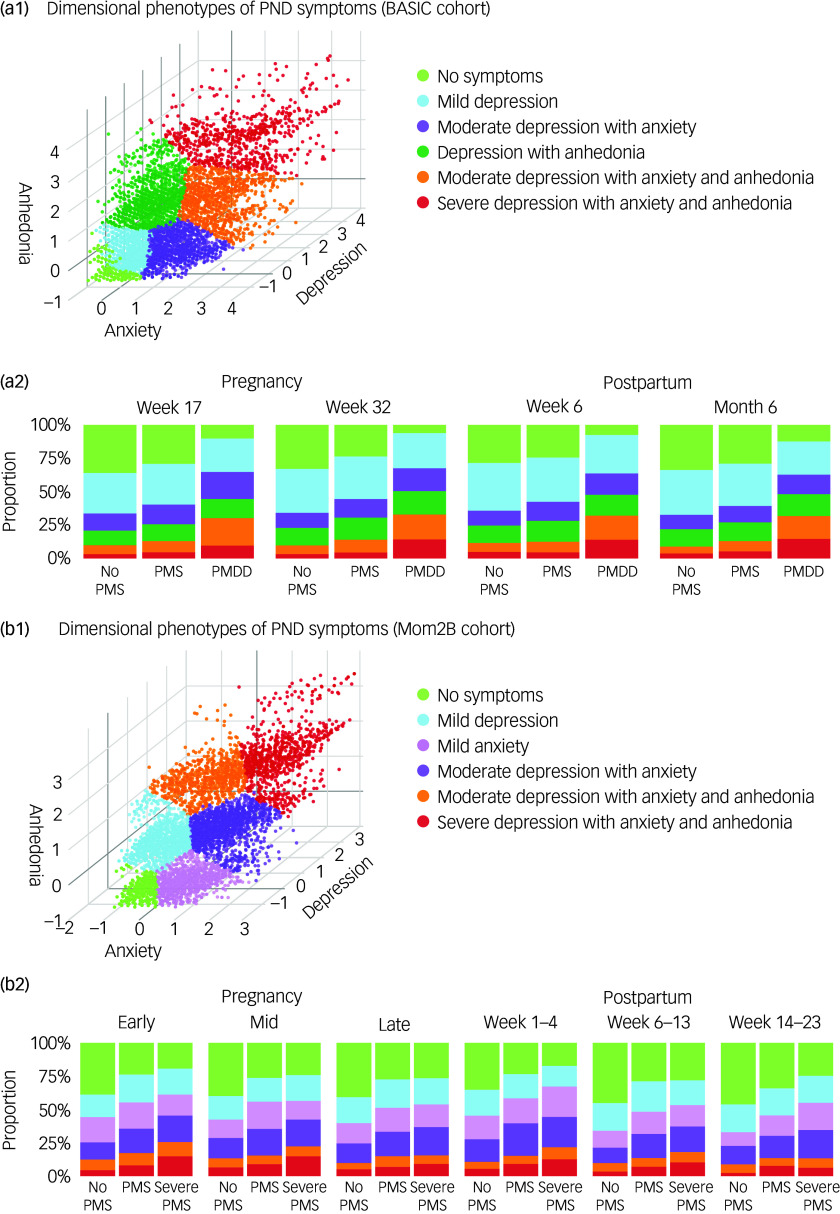
Dimensional phenotypes of perinatal depression (PND) symptoms and their association with premenstrual symptomatology over time. (a1) and (b1) 3D visualisation of the dimensional phenotypes of PND symptoms and severity for the BASIC and Mom2B cohorts respectively; (a2) and (b2) the proportions of the dimensional phenotypes of PND symptoms over time grouped by no premenstrual syndrome (No PMS), premenstrual syndrome (PMS) and premenstrual dysphoric disorder (PMDD) (BASIC)/severe PMS (Mom2B) for the BASIC and Mom2B cohorts respectively.

The early postpartum time point was associated with the highest number of PND cases of any severity in the BASIC (21%) and Mom2B cohorts (34%) (Fig. S1). Four severity groups were identified in the BASIC cohort, with the following proportions across the four time points: no depression (79–81%), mild to moderate depression (14–16%), moderate to severe (4.0–4.4%) and very severe (0.5–0.8%). Similarly, in the Mom2B cohort, four severity groups were identified, with the following proportions across the six time points: no depression (66–74%), mild to moderate (19–24%), moderate to severe (6.1–8.9%) and very severe (1.3–2.0%). Regarding symptom onset and persistence, three trajectories were identified in the BASIC cohort: gestational PND (9.3%), postpartum PND (13%) and persistent PND (2.1%). The same three trajectories based on symptom onset and persistence were identified in the Mom2B cohort: gestational PND (24%), postpartum PND (20%) and persistent PND (2.9%). Six symptom clusters based on three dimensions of PND symptoms, here referred to as dimensional phenotypes, were found in both the BASIC and the Mom2B cohorts (Fig. 2(a1) and (b1), Tables S4 and S5). Proportions of dimensional phenotypes of PND symptoms over time are presented in Fig. S2. Variables significantly associated with PND during the perinatal period in both cohorts are presented in the Supplementary Material.

### Association between PMS/PMDD and PND

Regarding PND symptom severity, differences in mean EPDS scores were noted at all time points in the BASIC cohort. On average, those reporting PMS and PMDD had approximately 1.2 and 1.7 times higher EPDS scores respectively compared with those reporting no premenstrual symptoms (Fig. 1(b1) and (b2)). In line, in the Mom2B cohort, those reporting PMS and severe PMS displayed 1.3 and 1.4 times higher EPDS scores on average compared with those reporting no PMS (Fig. 1(c1) and (c2)).

The highest proportion of PND among those reporting PMS and PMDD in the BASIC cohort was found in gestational week 32 (22.8 and 48.5% respectively). In the Mom2B cohort the greatest proportion of PND cases was noted in early postpartum and early pregnancy among those reporting PMS (37.7%) and severe PMS (43.1%). Post hoc analyses demonstrated that the differences were mainly driven by those reporting no PMS compared with PMDD at each time point, but were also present between those reporting no PMS in comparison with those reporting PMS, and those with PMS compared with PMDD, in the BASIC cohort. In the Mom2B cohort, the effect was driven by differences between those reporting PMS/severe PMS and no PMS. Differences in EPDS scores between those reporting severe PMS and PMS were noted only in early and mid-pregnancy.

Concerning clinically relevant diagnostic categorisation, PMS was reported by 29–34% of those with PND during pregnancy and postpartum in the BASIC cohort, compared with 58–65% in the Mom2B cohort. PMDD was reported by 15–17% of those with PND during pregnancy and postpartum in the BASIC cohort, whereas the corresponding proportion reporting severe PMS was 10–13% of those with PND in the Mom2B cohort. The proportions of those with PND among those reporting no PMS/PMS/PMDD remained similar between time points in the BASIC cohort, but varied slightly in the Mom2B cohort. The highest proportions of those reporting PMS were found in early pregnancy in the BASIC cohort, but in early postpartum in the Mom2B cohort, while the highest proportions of PMDD/severe PMS were found in early/mid-pregnancy in both cohorts (Table S3).

The odds of developing PND differed between those reporting no PMS, PMS or PMDD during the perinatal period. In the BASIC cohort, odds of PND were greater for those reporting PMS, and especially PMDD, with odds ratio 1.34–1.65 (95% CI 1.10–2.00) and odds ratio 2.31–3.05 (95% CI 1.66, 4.10) respectively (Table S6). In the Mom2B cohort, those reporting PMS had significantly higher odds for PND at all the postpartum time points, with odds ratio 1.37–1.68 (95% CI 1.03–2.29), whereas for severe PMS, only the mid-pregnancy time point was significant, with odds ratio 1.53 (95% CI 1.04–2.23) (Table S6).

No interaction effect between group and time was found in either cohort. In line with the observed associations at single time points between PMS/PMDD and PND in the BASIC cohort, a significant association was also found considering the whole perinatal period using the multivariable GEE models, clustered by participant and accounting for confounders, with odds ratio 1.63 (95% CI 1.47–1.82) and odds ratio 2.85 (95% CI 2.45–3.38) for those reporting PMS and PMDD respectively. In the Mom2B cohort, we found no significant associations between PMS/severe PMS and PND when considering the whole perinatal period; however, they were significant before adjustment for confounders.

**Fig. 3:**
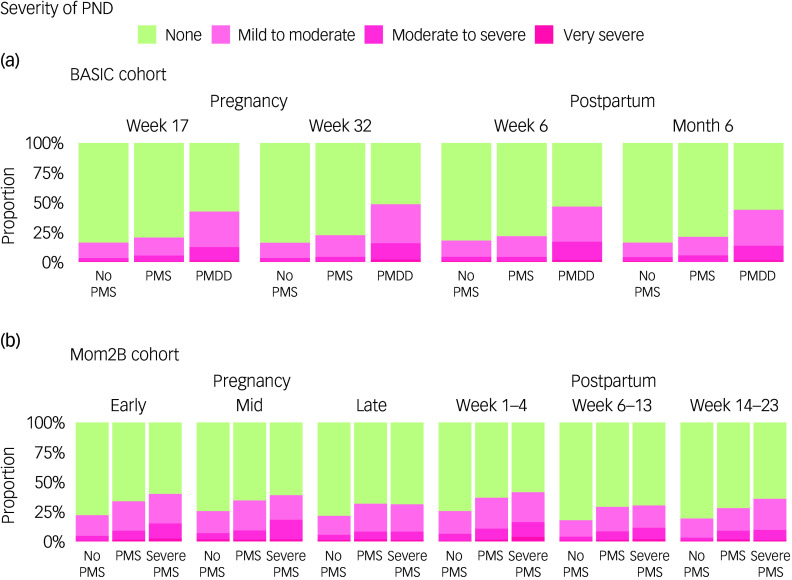
Proportions of perinatal depression (PND) severity over time in relation to premenstrual symptomatology. The distribution of PND severity categories during pregnancy and postpartum in (a) the BASIC cohort and (b) the Mom2B cohort. The plots are grouped by no premenstrual syndrome (No PMS), premenstrual syndrome (PMS) and premenstrual dysphoric disorder (PMDD) (BASIC)/severe PMS (Mom2B) for the BASIC and Mom2B cohorts respectively. PND severity was defined in terms of total score on the Edinburgh Postnatal Depression Scale: none (no PND), 0–9; mild to moderate, 10–15; moderate to severe, 16–21; and very severe, 22–30.

PND severity differed across those reporting no PMS, PMS and PMDD at all four time points in the BASIC cohort (Fig. 3(a), Table S7). The effect was mainly driven by the greater proportion of those reporting PMDD experiencing moderate to severe PND during pregnancy and postpartum week 6 (Table S8). The results were confirmed in the Mom2B cohort for all six time points through the perinatal period (Fig. 3(b), Table S9), and similarly the effect was mainly driven by the proportion of those reporting severe PMS experiencing moderate to severe PND in early and mid-pregnancy (Table S10).

### PMS or PMDD and propensity to develop a certain PND trajectory

**Fig. 4:**
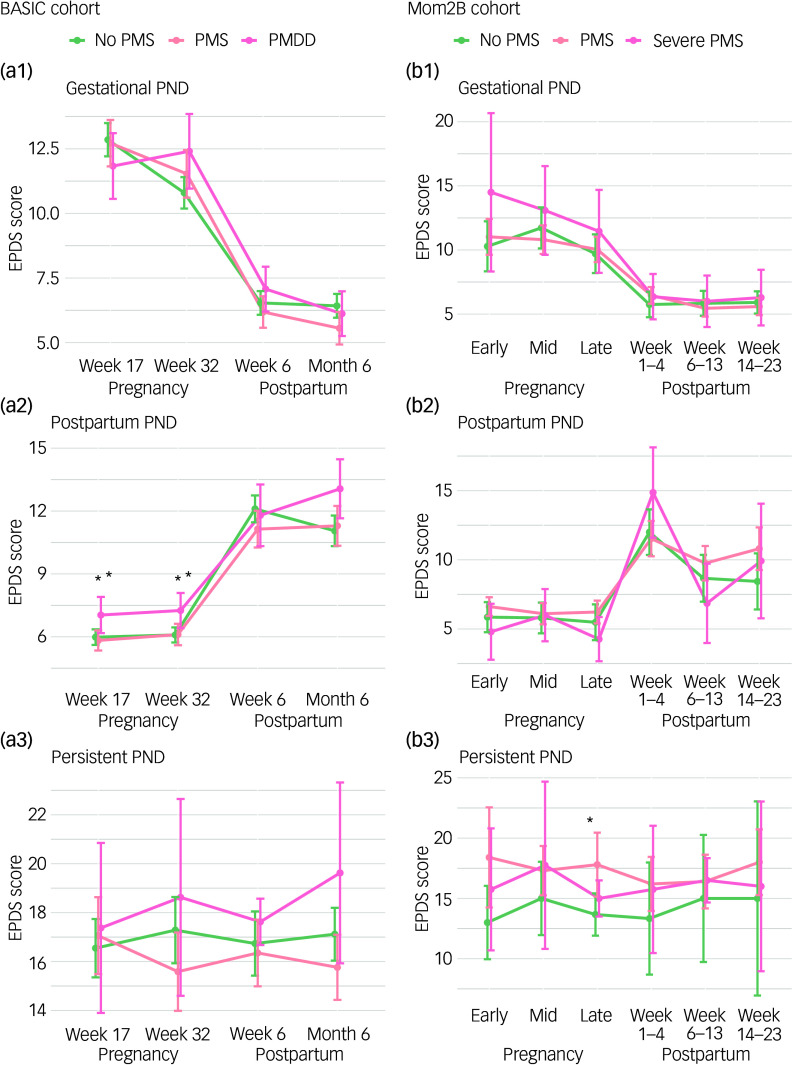
Symptom severity by perinatal depression (PND) trajectory in relation to premenstrual symptomatology. Mean Edinburgh Postnatal Depression Scale (EPDS) score plots of PND trajectories based on onset and persistence of symptoms. The trajectories are: gestational PND (EPDS ≥ 12 sometime during pregnancy); postpartum PND (EPDS ≥ 12 sometime postpartum); and persistent PND (EPDS ≥ 12 during both pregnancy and postpartum); trajectories are grouped on no premenstrual syndrome (No PMS), premenstrual syndrome (PMS) and premenstrual dysphoric disorder (PMDD) (BASIC)/severe PMS (Mom2B) for the BASIC (a1)–(a3) and Mom2B cohorts (b1)–(b3) respectively. The data points indicate mean values and the lines 95% confidence intervals. *P < 0.05.

Those reporting PMS in the BASIC cohort were more likely to develop the gestational PND trajectory compared with the control trajectory, while those reporting PMDD were more likely to develop any of the three PND trajectories, with gestational PND > postpartum PND > persistent PND in terms of effect size (Table S11). For those reporting PMS in the Mom2B cohort, no trajectory was significantly more common, while for those reporting severe PMS, the postpartum PND and particularly the persistent PND trajectories were more common (Table S12). In both cohorts, those reporting PMS and PMDD (BASIC)/severe PMS (Mom2B) displayed similar severity patterns over time for all PND symptom trajectories (gestational, postpartum and persistent PND) compared with those reporting no premenstrual symptoms (Fig. 4). The respective figures for controls with no PND are presented in the Supplementary Material in Fig. S3.

### Relationship between PMS or PMDD and dimensional phenotypes of PND symptoms

Greater proportions of dimensional phenotypes of PND symptoms, including symptoms of depressed mood, anxiety and anhedonia, were noted in those reporting PMS and PMDD (BASIC)/severe PMS (Mom2B) (Fig. 2(a2) and (b2), Tables S13 and S14). PMS and PMDD were significantly correlated with dimensional phenotypes of PND symptoms including moderate and severe symptoms. A significant overall difference between dimensional phenotypes of PND symptoms and premenstrual symptomatology was observed at all four time points in the BASIC (Table S15) and Mom2B cohorts (Table S16). In the BASIC cohort, the contribution of PMS, especially for ‘depression with anhedonia’, was elevated at week 32 of pregnancy, while the contribution of PMDD for ‘moderate depression with anxiety and anhedonia’ and ‘severe depression with anxiety and anhedonia’ was elevated at all time points, especially at weeks 17 and 32 of pregnancy respectively (Table S15). In the Mom2B cohort, the contributions of PMS were mostly elevated for ‘mild anxiety‘ at mid-pregnancy, while the contributions of severe PMS were particularly elevated for ‘severe depression with anxiety and anhedonia’ at early and mid-pregnancy (Table S16). Participants with no premenstrual symptoms were more represented among those with no PND. Furthermore, the proportions of each independent dimensional phenotype of PND symptoms between no PMS, PMS and PMDD in the BASIC cohort were significantly different at all time points. Several dimensional phenotypes of PND symptoms also significantly varied over time for PMS and PMDD (BASIC)/severe PMS (Mom2B). Proportions of dimensional phenotypes of PND symptoms in relation to severity categories of PND are further presented in Tables S17 and S18.

## Discussion

The association between two disorders linked to ovarian hormone sensitivity, namely PMS/PMDD and PND, was investigated in two large perinatal cohorts. The association between history of premenstrual symptomatology and PND was demonstrated for those reporting PMS, and even more strongly for PMDD, throughout the perinatal period, but especially during mid- to late pregnancy and early postpartum. Premenstrual symptomatology was associated with specific PND trajectories (based on onset and persistence of symptoms). Reports of PMS and PMDD were associated with dimensional phenotypes of PND symptoms, especially those manifested as severe and moderate symptoms of the dimensions depressed mood, anxiety and anhedonia. The findings were confirmed in two independent cohorts, one during 2009–2018 and the other during 2019–2023.

The present findings are consistent with the existence of a persistent (and potentially trait-like) ovarian hormone sensitivity throughout the female´s reproductive life. This is in line with previous observations, such as the qualitative study by Bloch et al^28^ and the majority of small retrospective studies.^7,29^ Expanding on the literature mostly focused on the postpartum period, the present findings indicate that those reporting PMS/PMDD also have a higher risk for depression during pregnancy and not only postpartum.

Regarding PMS/PMDD in relation to severity of PND symptoms, those reporting PMDD were more likely to develop moderate to severe PND (a score of 16–21 on the EPDS) during pregnancy and up to 6 months postpartum, which were the main contributors to the association. This finding further indicates that it is not only more common to have an EPDS score above the clinical cut-off (indicating PND) if a person has had PMDD, but also to experience more severe symptoms.

Concerning clinically relevant cut-offs for PND, a history of PMS, and especially PMDD, was associated with risk of PND at any time point during the perinatal period, with odds ratios between two and slightly above three for all time points in the BASIC cohort. It is noteworthy that these results were observed after accounting for covariates such as depression history, which is the most important known risk factor for PND, as well as antidepressant use during pregnancy and other relevant risk factors (the results remained virtually the same when excluding individuals with a history of depression and using antidepressants before or during pregnancy (Supplementary Material)). Nevertheless, it is possible that additional factors beyond hormone sensitivity contribute to a more general vulnerability. In the Mom2B cohort, the results were less prominent after accounting for covariates, and the association remained only postpartum for PMS and at a single time point during pregnancy for severe PMS. This was not surprising since the assessment of a history of PMDD and PMS in the BASIC cohort was, even though retrospective, based on DSM-5 and ICD-10 criteria respectively. Moreover, for the Mom2B cohort, it was assessed only using two yes/no questions, raising the importance of ICD-10/DSM-5-based assessment of PMS/PMDD in future studies.

The temporal occurrence and persistence of PND symptoms over time has been suggested to underly different trajectories of PND and their related risk factors. The postpartum trajectory was expected to be more common for those reporting PMS and PMDD, as it may be associated with progesterone withdrawal and other hormonal changes that occur after giving birth.^15,18^ This was found among those reporting severe PMS in the Mom2B cohort. In the BASIC cohort, the gestational PND trajectory was most common for those reporting PMS and PMDD, which is in line with previous evidence,^18^ but for those reporting PMDD, the postpartum trajectory was also more common compared with the control trajectory. However, we found no indication that the severity of the PND symptoms differed in each trajectory between those reporting no premenstrual symptoms, PMS and PMDD (BASIC)/severe PMS (Mom2B).

Inspired by RDoC and the work of Putnam et al,^20^ we investigated six dimensional phenotypes of PND symptoms based on three underlying symptom dimensions: depressed mood, anxiety and anhedonia. In both the BASIC and the Mom2B cohorts, the dimensional phenotypes of PND symptoms of higher severity were more prevalent among those reporting PMDD (BASIC)/severe PMS (Mom2B), particularly moderate and severe depression with anxiety and anhedonia during the whole perinatal period, notably during late pregnancy and early postpartum in most cases. This is the period most proximal to childbirth and most affected by hormonal fluctuations, corroborating the hypothesis of ovarian hormone sensitivity underlying PMS/PMDD and PND.^6,28,30^

The present findings thus indicate that sensitivity to ovarian hormone fluctuations can lead to mental distress during both the luteal phase of the menstrual cycle, such as in PMS and PMDD, as well as during pregnancy and postpartum, as in PND.

### Strengths and limitations

The strength of the current study is inherent in the longitudinal design and scrutiny of the contributors in pregnancy versus postpartum time points, as opposed to the DSM-5 approach of grouping pregnancy and postpartum together. Notable variables of interest, such as history of depression, parity and age, were considered as covariates. Remarkably, findings were virtually replicated in our two independent cohorts. Methodological considerations relating to the cohorts may contribute to explaining discrepancies. The Mom2B study included participants from all over Sweden, whereas the BASIC study included only those giving birth in the Uppsala catchment area of Sweden. Furthermore, Mom2B data were collected a decade after BASIC, when it had become more socially acceptable to speak out about mental health problems, possibly explaining the larger proportion reporting PMS in the Mom2B cohort. As the participants were not informed about the diagnostic criteria, potential confusion about the meaning of PMS (e.g. dysmenorrhea or heavy menstrual bleeding) could also be a contributing factor, although the term ‘severe PMS’ in the Mom2B cohort most likely also reflected treatment-seeking in addition to severity of symptoms. Participants in the ‘severe PMS’ group, on the other hand, most likely fulfilled the criteria for PMDD as this is the requirement in Sweden to receive treatment. Rather, it is probable that some of those with PMDD who had not received treatment, which happens for several reasons, might have been included in the PMS group. Additionally, Mom2B data were collected during and after the COVID-19 pandemic, potentially explaining the PND cluster of anxiety symptoms found in this cohort. The large size of both our samples is accompanied by the limitation of retrospectively assessed premenstrual symptoms, although the proportions seem in line with the literature.^4,5^ For BASIC, the participants were asked at gestational week 17 to answer 11 questions about premenstrual symptoms based on the DSM-5 A–D criteria, thus providing a more relevant assessment which was not affected by the current level of depression (Supplementary Material), whereas in the Mom2B cohort, the participants were asked at some point during pregnancy if they had ever experienced premenstrual symptoms or been treated for severe premenstrual symptoms. This highlights the need for a thorough assessment of PMDD.

### Clinical implications

Our study confirmed that sensitivity to ovarian hormone fluctuations manifested as symptoms of PMS and PMDD before pregnancy is associated with similar – or even worse – symptoms during pregnancy and the postpartum period. Therefore, clinicians should provide education during early pregnancy about the broader risks of the perinatal window, and encourage patients to contact their healthcare provider in the event of early warning symptoms of PND. It is important that counselling on the association of PMDD with PND is provided as part of routine prenatal care so that timely screening and tailored treatment plans can be implemented.

## Supporting information

Schleimann-Jensen et al. supplementary material Schleimann-Jensen et al. supplementary material

## Data Availability

The data that support the findings of this study are available from the corresponding author, E.C., on reasonable request. No study-specific analytic code was used.

## Supplementary material

The supplementary material is available online at https://doi.org/10.1192/bjp.2025.38
