# Supplementary material for: Trajectories and dimensional phenotypes of depressive symptoms throughout pregnancy and postpartum in relation to prior premenstrual symptoms
Source: Br J Psychiatry. 2025 Jun 20;226(6):401–9. doi: 10.1192/bjp.2025.38 (PMC12257281; doi:10.1192/bjp.2025.38)
Supplement: Schleimann-Jensen et al. supplementary material [file S0007125025000388sup001.docx]

**Trajectories and dimensional phenotypes of depressive symptoms throughout pregnancy and postpartum in relation to prior premenstrual symptoms**

Ella Schleimann-Jensen ^1^, Inger Sundström-Poromaa ^2^, Samantha Meltzer-Brody ^3^, Tory A. Eisenlohr-Moul ^4^, Fotis C Papadopoulos ^5^, Alkistis Skalkidou ^2^, Erika Comasco ^1^

## **Supplementary material**

**The BASIC study**

The Biology, Affect, Stress, Imaging and Cognition (BASIC) study is a population-based, prospective cohort study that enrolled 5492 females during pregnancy between September 2009 and November 2018 (1). The participants were followed through pregnancy up until one year postpartum. At gestational week 16-18 (early pregnancy) and 32 (mid-late pregnancy), the participants received an internet-based version of the Edinburgh Postnatal Depression Scale (EPDS) to fill in, as well as six weeks (early postpartum) and six months postpartum (late postpartum) (see Figure 1A). Apart from the EPDS questionnaire, the participants also received other questionnaires, at gestational week 16-18 they received questions about premenstrual symptoms designed by the researchers, but based on the DSM-5 criteria for PMDD (2), which is explained in this document. At gestational week 16-18 information about depressive and anxiety symptoms, medical information, lifestyle, sleep, trauma and stressful events, and sociodemographic information such as age, marital status, country of birth, education, employment was also collected. At gestational week 32 information about depressive and anxiety symptoms, pregnancy complications, medical information, lifestyle, sleep, and psychological measures was collected. At week six postpartum information about the delivery, the delivery experience, lifestyle, sleep, psychological measures, trauma and stressful events, partner support, and breastfeeding was collected. At six months postpartum information about depressive and anxiety symptoms, medical information, lifestyle, sleep, psychological measures, partner support, and breastfeeding was collected. At one-year postpartum additional information about medical information, sleep, psychological measures, trauma and stressful events, including The Lifetime Incidence of Traumatic Events (LITE) questionnaire, considering traumatic events up to the age of 18 (3), and breastfeeding was collected.

**The Mom2B study**

The Mom2B is a smartphone-based application targeting all Swedish-speaking females over 18 years who are pregnant or up to three months postpartum (4). The study has been going on since 2019, and is still on going with the goal to collect data from at least 5000 females with complete outcome measures. Mom2B was created with the aim to use the digital data from the application to develop prediction models that could be used to find females high risk for pregnancy and postpartum complications at an early stage. In the Mom2B app the females fill surveys and questionnaires at several timepoints during the perinatal period. The Edinburgh Postnatal Depression Scale (EPDS) is the primary outcome, and is filled by the females at three timepoints during pregnancy; week 12-22 (early pregnancy), week 24-34 (mid pregnancy), and week 36-42 (late pregnancy), as well as six timepoints postpartum; week 1-4 (early postpartum), week 6-13 (mid postpartum), week 14-23 (late postpartum), week 24-35, week 36-49, and week 50-52 (see Figure 1A). Although, only the first three postpartum timepoints were used in the analyses due to large amounts of missing data in the last three timepoints. Information about previous premenstrual symptomatology is collected anytime during pregnancy with the two questions “Did you ever experience PMS?” (yes/no), and “Have you ever been treated for severe PMS?” (yes/no). Other information about stress, general health, personality, attachment, parenthood, psychiatric history, psychological health, lifestyle, perinatal experiences, and relationships are also collected at different timepoints throughout the perinatal period via the application. Amongst these, The Lifetime Incidence of Traumatic Events (LITE) questionnaire, considering traumatic events up to the age of 18 (3), is included.

**The EPDS questionnaire**

The Edinburgh Postnatal Depression Scale (EPDS) was used in the BASIC and is used in the Mom2B project to assess depressive symptoms during the perinatal period. The EPDS consists of the following ten items which are all rated on a scale from 0 to 3:
1) “I have been able to laugh and see the funny side of things”,
2) “I have looked forward with enjoyment to things”,
3) “I have blamed myself unnecessarily when things went wrong”,
4) “I have been anxious or worried for no good reason”,
5) “I have felt scared or panicky for no good reason”,
6) “Things have been getting on top of me”,
7) “I have been so unhappy that I have had difficulty sleeping”,
8) “I have felt sad or miserable”,
9) “I have been so unhappy that I have been crying”,
10) “The thought of harming myself has occurred to me” (5).

**The DSM-5 A-D and F criteria for PMDD**

According to criteria A-D of the DSM-5, participants in the BASIC cohort reported on the experience of any of the following symptoms during the premenstrual phase: 1)“Depressed mood or hopelessness”, 2)“Anxiety or tension”, 3)“Affective lability”, 4)“Irritability or anger”, 5)“Decreased interest in usual activities”, 6)“Concentration difficulties”, 7)“Marked lack of energy”, 8)“Marked change in appetite, overeating or food cravings”, 9)“Hypersomnia or insomnia”, 10)“Feeling overwhelmed or out of control”, 11)”Other physical symptoms”. The participant had to report on having experienced at least five of the symptoms, where at least one was a core symptom (symptom 1-4). In addition to this, they had to experience the symptoms in each menstrual cycle and the symptoms needed to cause clinically significant distress or interfere with the daily life of the participant (2). The DSM-5 criteria F further describes that these symptoms must have been reported in a daily diary for at least two menstrual cycles which was not done in this particular study, since the premenstrual symptomatology was reported retrospectively at one timepoint.

**The ICD-10 criteria for PMS**

The ICD-10 criteria for PMS require at least one symptom arising around the onset of menses each month, and it was used in the BASIC cohort to confirm PMS. The symptoms could be psychological, and/or physical with symptoms such as bloating, weight gain, breast tenderness, swelling or pain, but can also include concentration difficulties, and sleep or appetite disturbances (6).

**Severity categories and trajectories of PND**

Participants were assigned to four severity categories; no depression (0-9 EPDS points), mild-moderate (10-15 EPDS points), moderate-severe (16-21 EPDS points), and very severe (22-30 EPDS points) (20) at each of the four timepoints. PND trajectories based on onset and persistency of symptoms, were assessed by categorizing the subjects into four groups: controls (EPDS < 12 during the whole perinatal period), gestational PND (depression only during pregnancy; EPDS ≥ 12 at least at one timepoint during pregnancy and EPDS < 12 at all timepoints postpartum), postpartum PND (depression only during the postpartum period; EPDS ≥ 12 at least at one timepoint during postpartum and EPDS < 12 at all timepoints during pregnancy), and persistent PND (depression throughout pregnancy and postpartum; EPDS ≥ 12 at all timepoints during pregnancy and postpartum). The criteria for the PND trajectories lead to small sample sizes for some of the groups due to missing data at some timepoints for several participants, particularly for persistent PND in the Mom2B cohort.

**The Research Domain Criteria (RDoC)**

The Research Domain Criteria (RDoC) was launched in 2009 in order to acquire a deeper understanding of the nature of mental disorders, with the goal to approach research differently which could lead to better diagnosis, prevention, intervention and cures (7). Today it consists of six major functional domains, which explains different types of neurobehavioral functioning, and constitutes the base of the framework. Each domain includes aspects represented by both psychological and biological dimensions. Amongst the dimensions are arousal and negative valence, which are connected to sensitivity to stimuli and anxiety, and which we, inspired by Putnam et al. (8), related to the dimensional phenotypes in this particular study, since anxiety is one of the most common symptoms of PND and PMDD.

**Participant characteristics**

Information on race and ethnicity of the study subjects was not collected; the sample merely included a group of Caucasian individuals based on national statistics from Sweden. PPD, PMS and PMDD seem to be universally prevalent; however, findings may not generalize to non-White participants. Furthermore, because of the nature of the study, only females assigned at birth were included. The population included in the BASIC and Mom2B has been described in (1) and (4), respectively. Both studies are associated with an ethics permit, written informed consent has been obtained from participants after the procedure(s) had been and adequate protections in place to ensure patient confidentiality have been implemented (1, 4).

**Statistical analysis**

**Associations with PND**

There were a number of variables significantly associated with PND during the perinatal period in both cohorts, including lower maternal age, depression-history, family history of psychiatric disorders, more traumatic interpersonal events before the age of 18, antidepressant-use before or during pregnancy, psychiatric treatment, any psychiatric or somatic diagnosis before or during pregnancy, breastfeeding, poor partner-support and pregnancy or postpartum complications.

**Factor analysis and k-means clustering**

Dimensional constructs were computed based on the ten EPDS items from each participant and each timepoint. Barlett’s test of sphericity and the Kaiser-Meyer-Olkin measure were used to test the sphericity and the sampling adequacy of the data respectively. A factor analysis was run with an oblimin rotation since correlation between some of the variables was expected. The whole dataset was used as the sampling adequacy for factor analysis was only appropriate when including all participants. The number of factors were first set to ten as the number of EPDS questions are ten. The final number of factors were then set to the number of factors with an eigenvalue > 1, which were three. The factor analysis was run with a final number of factors of 3 (depressed mood, anxiety and anhedonia), separately for both the BASIC and Mom2B cohort. Barlett’s test of sphericity showed significant correlation between the variables to run a factor analysis (p = 2.22*10^-16^) and Kaisers test showed that the sampling adequacy was good (KMO = 0.90 for BASIC, KMO = 0.91 for Mom2B). These values were obtained when including all participants in the factor analysis. The factor loadings confirmed that there are three underlying factors of the EPDS questions; “Depressed mood”, “Anxiety” and “Anhedonia”, see Table S4 and Table S5 for the BASIC and Mom2B respectively. Running the analysis separately per timepoint generated similar results.

Continuously, clustering using the unsupervised k-means method was based on the three dimensions obtained from the factor analysis. K-means clustering is based on an iterative process in which a number of *k* clusters are created based on minimizing the distance to *k* differentially placed centroids to find the most optimal clusters with each data point only belonging to one cluster. Clusters obtained by choosing k-values between k = 2 to k = 10 were compared. Based on previous literature (8) and the elbow method, k = 6 was chosen for both the BASIC and Mom2B as the most appropriate number of clusters. Six clusters were found in both cohorts, with five of them being defined the same in both cohorts; “no PND”, “mild depression”, “moderate depression with anxiety”, “moderate depression with anxiety and anhedonia”, and “severe depression with anxiety and anhedonia”. One cluster was defined differently between the two cohorts, which was “depression with anhedonia” for BASIC and “mild anxiety” for Mom2B. This was based on the location of the centroids in the 3D space. The cluster “no PND” was found since all participants were included in the analysis, in contrast to Putnam et al (8). The clusters are visualized in 3D in Figure 3A1 and B1 for the BASIC and Mom2B cohorts respectively. Each participant was included in one cluster at each timepoint, which is visualized in Figure S2A and B for the BASIC and Mom2B respectively. These clusters were then related to premenstrual symptomatology which is visualized in Figure 2A2 and B2 for the BASIC and Mom2B cohorts respectively.

**Extra analyses**

**Excluding participants with a history of depression**

History of depression is one of the most important risk factors known for PND. The variable was included in the models as a covariate, but some extra analyses when excluding individuals reporting a history of depression were also performed. When excluding the individuals with a history of depression, the mean EPDS scores for those reporting no premenstrual symptomatology is around 4-5 during the whole time period in both the BASIC and Mom2B cohorts, while for those reporting PMS it is around 5-6 in both the Mom2B and BASIC cohorts, and for those reporting PMDD in the BASIC cohort it is around 8-9, but for those reporting severe PMS in the Mom2B cohort it is between 4-8, differing more between the different timepoints. In the BASIC cohort this is in line with the whole sample, although in Mom2B it differs a bit from the whole sample. The associations between PMS and PND although remained significant in the BASIC cohort at all timepoints, as well as the associations between PMDD and PND, even after adjustment for other confounders. In Mom2B the postpartum timepoints week 6-13 and week 14-23 were significant after adjustment for other confounders for PMS, while for severe PMS, the late pregnancy timepoint (week 36-42), and postpartum timepoints week 1-4 and week 6-13 were significant after adjustment for confounders. For the BASIC cohort this was again in line with the results from the whole sample, while the results differed in Mom2B compared to the whole sample, especially for severe PMS.

**Excluding controls using SSRIs during pregnancy**

When excluding individuals without PND and using SSRIs during pregnancy, the mean EPDS scores remains approximately the same for each group; no PMS, PMS and PMDD/severe PMS at both week 17 of pregnancy and week 32 of pregnancy in BASIC, and at week 12-22, 24-34 and 36-42 of pregnancy in Mom2B. The associations between PND and PMS and PMDD/severe PMS also remained significant at all timepoints during pregnancy in both the BASIC and Mom2B cohort.

**Bias of the PMDD assessment being at one of the timepoints using EPDS**

Chi-square tests and correlation analyses were run in order to investigate whether the depression level at the timepoint of the PMS/PMDD assessment in the BASIC cohort (week 16-18 of pregnancy) could have affected the results. No elevated correlation between depression score at week 17 of pregnancy and PMS/PMDD was found in the correlation analyses, and no indication in the chi-square tests. The extra analyses regarding this potential bias was only performed for the data from the BASIC cohort, and not the data from the Mom2B cohort, since in the Mom2B cohort the PMS/PMDD assessment was not performed at any specific timepoint, but at any timepoint during pregnancy.

**Supplementary figures**

**
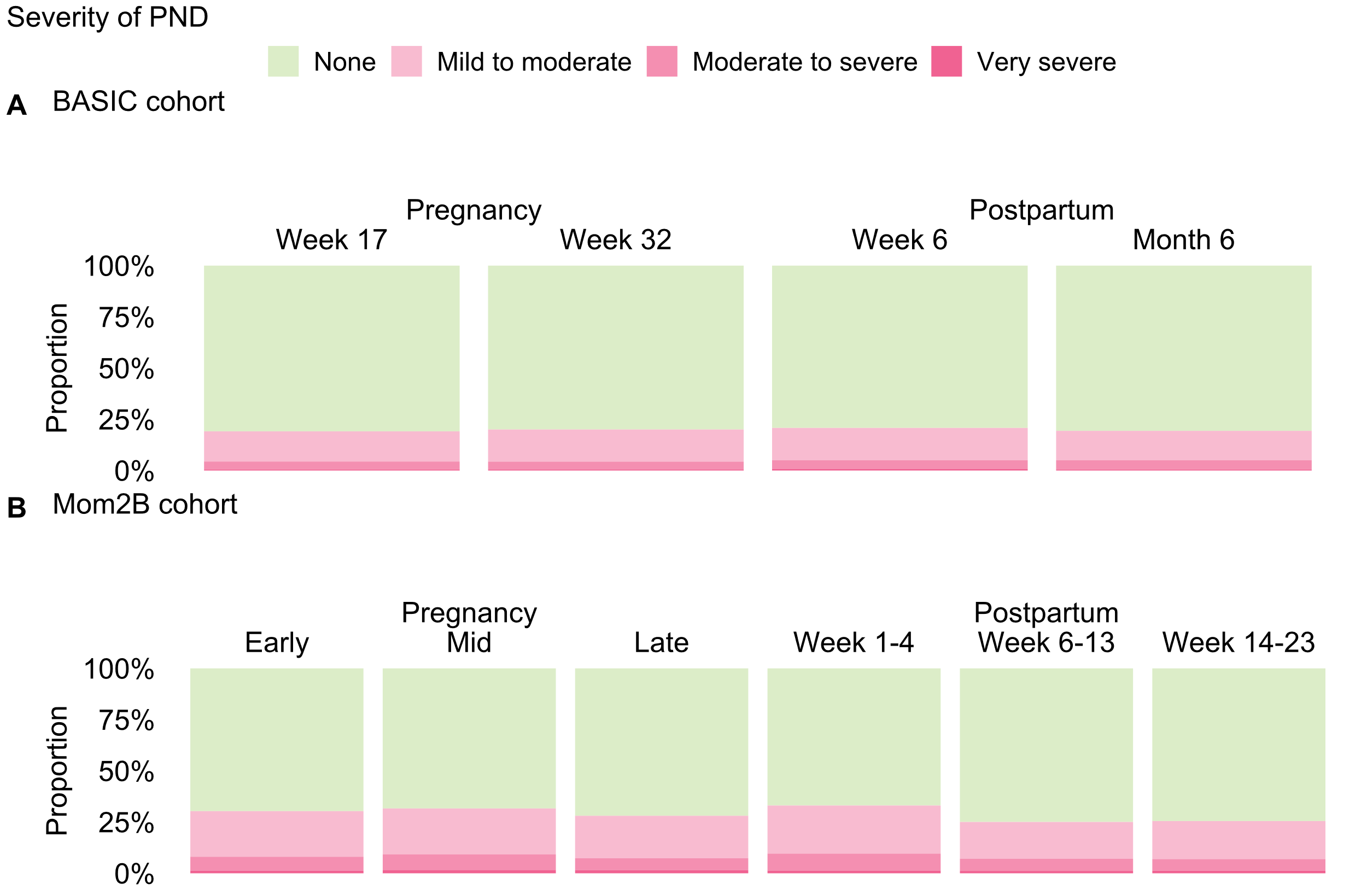
**

**Figure S1. Proportions of PND severity over time.** The distribution of PND severity categories during pregnancy and postpartum in BASIC (A) and Mom2B (B). None corresponds to 0-9p in total EPDS score, Mild to moderate 10-15p, Moderate to severe 16-21p and Very severe 22-30p.


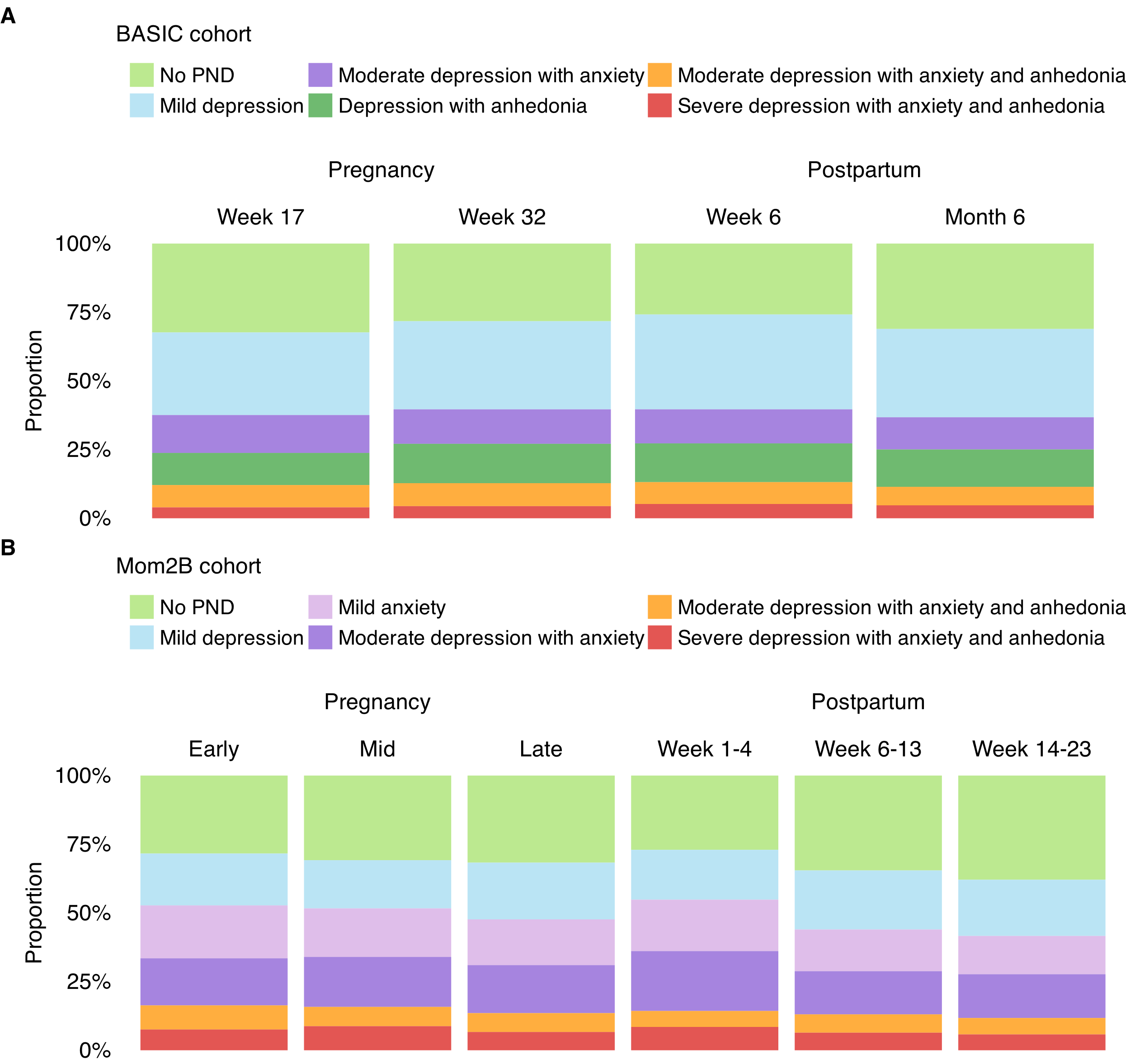


**Figure S2.** **Proportions of dimensional phenotypes of PND over time.** Plot A shows the proportions of the six dimensional phenotypes of PND symptomatology over time found in the BASIC cohort using factor analysis and k-means clustering. Plot B shows the proportions for the six dimensional phenotypes found in the Mom2B cohort.

**
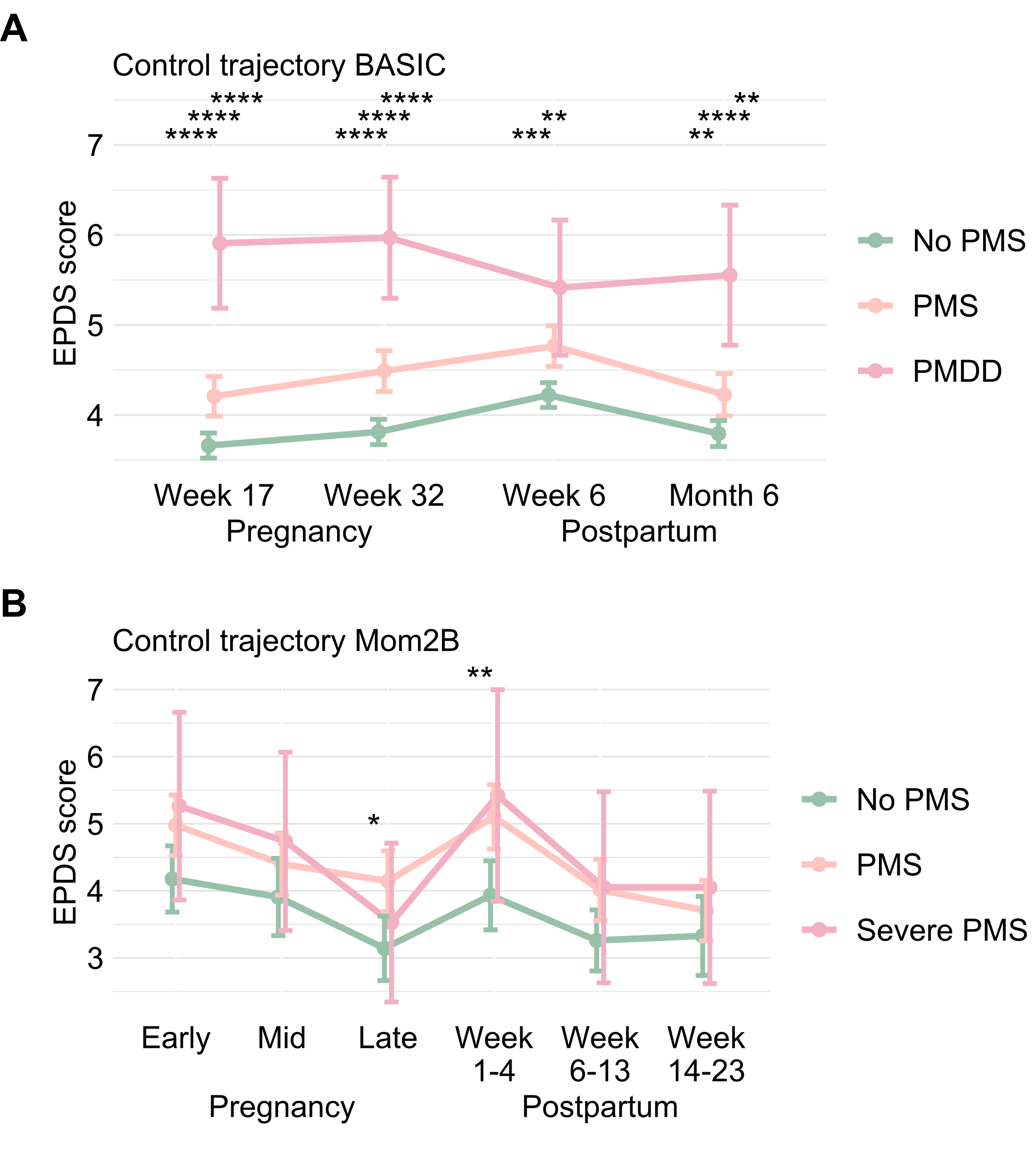
**

**Figure S3. Symptoms severity in the PND control trajectory in relation with premenstrual symptomatology.** Mean plots of the control trajectory for those without PND, with EPDS < 12 at all timepoints. Plot A shows the control trajectory for BASIC and plot B shows the control trajectory for Mom2B. The continuous EPDS score of each trajectory was compared between the groups no PMS, PMS and PMDD (BASIC)/severe PMS (Mom2B) using the Kruskal-Wallis non-parametric test, applying Dunn’s test for multiple comparisons for significant results. Results with ‘*’ indicate p<0.05, and those with ‘**’ indicate p≤0.01, and those with ‘***’ indicate p≤0.001, and those with ‘****’ indicate p≤0.0001.

**Supplementary tables**

**Table S1. Participants characteristics in BASIC.**

|  | No PMS/PMDD N = 2908 | PMS (ICD criteria) N = 1367 | PMDD (DSM-V) N = 294 | |
| --- | --- | --- | --- | --- |
| **EPDS pregnancy week 17 (Mean (SD))** | 5.3 (4.5) | 6.3 (4.8) | 9.3 (5.2) |  |
| **EPDS pregnancy week 32 (Mean (SD))** | 5.4 (4.4) | 6.4 (4.6) | 9.8 (5.2) |  |
| **EPDS postpartum week 6 (Mean (SD))** | 5.8 (4.6) | 6.5 (4.5) | 9.5 (5.4) |  |
| **EPDS postpartum month 6 (Mean (SD))** | 5.3 (4.6) | 6.1 (4.8) | 9.3 (5.6) |  |
| **Maternal age (Mean (SD))** | 31.6 (4.5) | 31.4 (4.6) | 31.3 (4.5) |  |
| **Psychiatric history (N (%))** |  |  |  |  |
| *No* | 2071 (71 %) | 913 (67 %) | 117 (40 %) |  |
| *Yes* | 798 (27 %) | 448 (33 %) | 177 (60 %) |  |
| **Primiparas (N (%))** | 1387 (48 %) | 665 (49 %) | 123 (42 %) |  |
| **Multiparas (N (%))** | 1373 (47 %) | 633 (46 %) | 153 (52 %) |  |
| **Employment (N (%))** |  |  |  |  |
| *Employed* | 2616 (90 %) | 1223 (90 %) | 251 (85 %) |  |
| *Unemployed* | 77 (2.6 %) | 38 (2.8 %) | 14 (4.8 %) |  |
| *Studying* | 207 (7.1 %) | 101 (7.4 %) | 28 (9.5 %) |  |
| **Education (N (%))** |  |  |  |  |
| *University* | 2262 (78 %) | 1004 (73 %) | 214 (73 %) |  |
| *Else* | 632 (22 %) | 356 (26 %) | 80 (27 %) |  |
| **Romantic relationship (N (%))** |  |  |  |  |
| *Married/Co-habiting* | 1329 (46 %) | 625 (46 %) | 132 (45 %) |  |
| *Divorced/Unmarried* | 1302 (45 %) | 604 (44 %) | 121 (41 %) |  |
| **Pregnancy upon fertility treatment (N (%))** |  |  |  |  |
| *No* | 2572 (88 %) | 1230 (90 %) | 259 (88 %) |  |
| *Yes* | 324 (11 %) | 137 (10 %) | 35 (12 %) |  |
| **Family history of psychiatric disorders (N (%))** |  |  |  |  |
| *No* | 1960 (67 %) | 881 (64 %) | 147 (50 %) |  |
| *Yes* | 948 (33 %) | 486 (36 %) | 147 (50 %) |  |
| **Pharmacological treatment (N (%))** |  |  |  |  |
| 1 year before pregnancy |  |  |  |  |
| Antidepressants |  |  |  |  |
| *No* | 2683 (92 %) | 1230 (90 %) | 225 (77 %) |  |
| *Yes* | 225 (7.7 %) | 137 (10 %) | 69 (24 %) |  |
| Hormone treatment |  |  |  |  |
| *No* | 1844 (63 %) | 905 (66 %) | 203 (69 %) |  |
| *Yes* | 1064 (37 %) | 442 (34 %) | 91 (31 %) |  |
| Other |  |  |  |  |
| *No* | 2677 (92 %) | 1245 (91 %) | 234 (80 %) |  |
| *Yes* | 231 (7.9 %) | 122 (8.9 %) | 60 (20 %) |  |
| During pregnancy |  |  |  |  |
| Antidepressants |  |  |  |  |
| *No* | 2766 (95 %) | 1286 (94 %) | 259 (88 %) |  |
| *Yes* | 142 (4.9 %) | 81 (5.9 %) | 35 (12 %) |  |
| Hormone treatment |  |  |  |  |
| *No* | 2534 (87 %) | 1198 (88 %) | 250 (85 %) |  |
| *Yes* | 374 (13 %) | 169 (12 %) | 44 (15 %) |  |
| Other |  |  |  |  |
| *No* | 2807 (97 %) | 1319 (97 %) | 271 (92 %) |  |
| *Yes* | 101 (3.5 %) | 48 (3.5 %) | 23 (7.8 %) |  |
| **Psychological treatment (N (%))** |  |  |  |  |
| *No* | 1476 (51 %) | 593 (43 %) | 67 (23 %) |  |
| *Yes* | 1432 (49 %) | 774 (57 %) | 227 (77 %) |  |
| **Registry-based diagnosis (N (%))*** |  |  |  |  |
| 1 year before pregnancy |  |  |  |  |
| *No* | 2287 (79 %) | 1071 (78 %) | 205 (70 %) |  |
| *Yes* | 621 (21 %) | 296 (22 %) | 89 (30 %) |  |
| During pregnancy |  |  |  |  |
| *No* | 1136 (39 %) | 474 (35 %) | 94 (32 %) |  |
| *Yes* | 1772 (61 %) | 893 (65 %) | 200 (68 %) |  |
| **Early life stressors (Mean (SD))** |  |  |  |  |
| *Interpersonal events* | 0.4 (0.8) | 0.5 (1) | 0.7 (1.4) |  |
| *Non-interpersonal events* | 0.7 (1.2) | 0.8 (1.3) | 0.9 (1.4) |  |
| **Breastfeeding (N (%))** |  |  |  |  |
| 6 weeks postpartum |  |  |  |  |
| *No* | 181 (6.2 %) | 101 (7.4 %) | 22 (7.5 %) |  |
| *Yes* | 2436 (84 %) | 1121 (82 %) | 238 (81 %) |  |
| 6 months postpartum |  |  |  |  |
| *No* | 577 (20 %) | 290 (21 %) | 67 (23 %) |  |
| *Yes* | 1918 (66 %) | 872 (64 %) | 175 (60 %) |  |
| **Partner support (N (%))** |  |  |  |  |
| 6 weeks postpartum |  |  |  |  |
| *No* | 73 (2.5 %) | 47 (3.4 %) | 8 (2.7 %) |  |
| *Yes* | 2530 (87 %) | 1168 (85 %) | 249 (85 %) |  |
| 6 months postpartum |  |  |  |  |
| *No* | 81 (2.8 %) | 47 (3.4 %) | 12 (4.1 %) |  |
| *Yes* | 2398 (83 %) | 1103 (81 %) | 229 (78 %) |  |
| **Pregnancy complications (N (%))** |  |  |  |  |
| *No* | 849 (29 %) | 378 (28 %) | 56 (19 %) |  |
| *Yes* | 1883 (65 %) | 901 (66 %) | 218 (74 %) |  |
| **Postpartum complications (N (%))** |  |  |  |  |
| *No* | 2153 (74 %) | 1005 (74 %) | 203 (69 %) |  |
| *Yes* | 489 (17 %) | 229 (17 %) | 62 (21 %) |  |
|  |  |  |  |  |
|  |  |  |  |  |
|  |  |  |  |  |

* Registry-based diagnosis refers to any type of diagnosis, including obstetric, psychiatric, somatic and metabolic diagnoses.

**Table S2. Participants characteristics in Mom2B.**

|  | No PMS  N = 1642 | PMS N = 2539 | Severe PMS  N = 410 | |
| --- | --- | --- | --- | --- |
| **EPDS pregnancy week 12-22** | 6.4 (4.9) | 8.2 (5.2) | 9.3 (5.9) | |
| **EPDS pregnancy week 24-34** | 6.7 (5.6) | 8.1 (5.5) | 9.3 (6.0) | |
| **EPDS pregnancy week 36-42** | 6.0 (5.3) | 7.6 (5.3) | 8.1 (5.3) | |
| **EPDS postpartum week 1-4** | 6.7 (5.1) | 8.4 (5.4) | 9.3 (5.7) | |
| **EPDS postpartum week 6-13** | 5.8 (4.7) | 7.5 (5.2) | 8.3 (5.6) | |
| **EPDS postpartum week 14-23** | 5.6 (4.8) | 7.3 (5.4) | 8.1 (5.0) | |
| **Age (Mean (SD))** | 31.3 (4.5) | 31.4 (4.4) | 31.4 (4.5) | |
| **Psychiatric history (N (%))** | 715 (44 %) | 1530 (60 %) | 282 (69 %) | |
| **Primiparas (N (%))** | 798 (49 %) | 1312 (52 %) | 196 (48 %) | |
| **Multiparas (N (%))** | 791 (48 %) | 1158 (46 %) | 202 (49 %) | |
| **Employment (N (%))** |  |  |  | |
| *Full time employed* | 818 (50 %) | 1233 (49 %) | 188 (46 %) | |
| *Part time employed* | 155 (9.4 %) | 233 (9.2 %) | 45 (11 %) | |
| *Studying* | 58 (3.5 %) | 90 (3.5 %) | 19 (4.6 %) | |
| *Unemployed* | 14 (0.85 %) | 20 (0.79 %) | 4 (0.98 %) | |
| **Education (N (%))** |  |  |  | |
| *Else* | 510 (31 %) | 741 (29 %) | 130 (32 %) | |
| *University* | 1079 (66 %) | 1727 (68 %) | 265 (65 %) | |
| **Romantic relationship (N (%))** |  |  |  | |
| *Living with partner* | 1520 (93 %) | 2354 (93 %) | 374 (91 %) | |
| *Partner but not living together* | 27 (1.6 %) | 56 (2.2 %) | 13 (3.2 %) | |
| *Single* | 39 (2.4 %) | 54 (2.1 %) | 9 (2.2 %) | |
| **Pregnancy upon fertility treatment (N (%))** | 165 (10 %) | 221 (8.7 %) | 41 (10 %) | |
| **Pharmacological treatment (N (%))** |  |  |  | |
| Antidepressants |  |  |  | |
| *No* | 367 (22 %) | 559 (22 %) | 68 (17 %) | |
| *During pregnancy* | 96 (5.8 %) | 230 (9.1 %) | 66 (16 %) | |
| *Early postpartum* | 8 (0.49 %) | 6 (0.24 %) | 3 (0.73 %) | |
| *Late postpartum* | 7 (0.43 %) | 5 (0.19 %) | 2 (0.49 %) | |
| Other treatment |  |  |  | |
| *No* | 212 (13 %) | 318 (13 %) | 40 (9.8 %) | |
| *During pregnancy* | 440 (27 %) | 784 (31 %) | 141 (34 %) | |
| *Early postpartum* | 1 (0.06 %) | 4 (0.16 %) | 0 (0 %) | |
| *Late postpartum* | 19 (1.2 %) | 26 (1.0 %) | 7 (1.7 %) | |
| **Psychological treatment (N (%))** |  |  |  | |
| *No* | 199 (12 %) | 252 (9.9 %) | 28 (6.8 %) | |
| *During pregnancy* | 105 (6.4 %) | 232 (9.1 %) | 47 (11 %) | |
| *Postpartum* | 20 (1.2 %) | 55 (2.2 %) | 7 (1.7 %) | |
| **Early life stressors (before the age of 18) (Mean (SD))** |  |  |  | |
| *Total events* | 2.37 (2.02) | 2.95 (2.32) | 3.27 (2.40) | |
| *Interpersonal events* | 0.86 (1.11) | 1.13 (1.31) | 1.31 (1.43) | |
| *Non-interpersonal events* | 1.39 (1.28) | 1.63 (1.36) | | 1.73 (1.37) |
| **Breastfeeding (N (%))** |  |  |  | |
| *No* | 18 (1.1 %) | 30 (1.1 %) | 4 (0.98 %) | |
| *Early postpartum* | 169 (10 %) | 283 (11 %) | 45 (11 %) | |
| *Late postpartum* | 150 (9.1 %) | 235 (9.3 %) | 25 (6.1 %) | |
| **Partner support early postpartum (N (%))** | 594 (36 %) | 908 (36 %) | 129 (31 %) | |
| **Partner support late postpartum (N (%))** | 336 (20 %) | 516 (20 %) | 63 (15 %) | |
| **Pregnancy complications (N (%))** | 1234 (75 %) | 2052 (81 %) | 327 (80 %) | |
|  |  |  |  | |
|  |  |  |  | |
|  |  |  |  | |

**Table S3.** **PND proportions by group and timepoint.** The total proportion of PND at each timepoint, and proportions of those with PND reporting no PMS, PMS or PMDD (BASIC)/severe PMS (Mom2B) at each timepoint. The percentages are based on the total number of individuals in each group.

|  | Pregnancy | | | | Postpartum | | | |  |
| --- | --- | --- | --- | --- | --- | --- | --- | --- | --- |
| **BASIC cohort** | Week 17 | | Week 32 | | Week 6 | | Month 6 | |  |
| Total | 13 % | | 13 % | | 14 % | | 12 % | |  |
| No PMS | 10 % | | 10 % | | 12 % | | 10 % | |  |
| PMS | 15 % | | 14 % | | 13 % | | 14 % | |  |
| PMDD | 32 % | | 34 % | | 33 % | | 32 % | |  |
| **Mom2B cohort** | Week 12-22 | Week 24-34 | | Week 36-42 | Week 1-4 | Week 6-13 | | Week 14-23 | |
| Total | 22 % | 24 % | | 20 % | 24 % | 20 % | | 20 % | |
| No PMS | 16 % | 19 % | | 14 % | 17 % | 13 % | | 14 % | |
| PMS | 24 % | 25 % | | 22 % | 26 % | 21 % | | 23 % | |
| Severe PMS | 34 % | 34 % | | 25 % | 31 % | 26 % | | 25 % | |

**Table S4. Factor loadings from the factor analysis in BASIC.** The factor loadings are presented for each EPDS question in each of the three dimensions found through factor analysis of the EPDS scores from each of the four timepoints. The bold values are values > 0.4 which are contributing mostly to the factor.

| EPDS question | Depressed mood | Anxiety | Anhedonia |
| --- | --- | --- | --- |
| (1) I have been able to laugh and see the funny side of things. | 0.24 | -0.03 | **0.67** |
| (2) I have looked forward with enjoyment to things. | -0.05 | 0.05 | **0.88** |
| (3) I have blamed myself unnecessarily when things went wrong. | **0.48** | 0.3 | -0.10 |
| (4) I have been anxious or worried for no good reason. | 0.15 | **0.62** | -0.03 |
| (5) I have felt scared or panicky for no good reason. | -0.04 | **0.80** | 0.06 |
| (6) Things have been getting to me. | **0.51** | 0.06 | 0.09 |
| (7) I have been so unhappy that I have had difficulty sleeping. | **0.43** | 0.22 | 0.14 |
| (8) I have felt sad or miserable. | **0.78** | -0.02 | 0.11 |
| (9) I have been so unhappy that I have been crying. | **0.75** | 0 | -0.02 |
| (10) The thought of harming myself has occurred to me. | 0.19 | 0.19 | 0.08 |

**Table S5. Factor loadings from the factor analysis in Mom2B.** The factor loadings are presented for each EPDS question in each of the three dimensions found through factor analysis of the EPDS scores from each of the six timepoints. The bold values are values > 0.4 which are contributing mostly to the factor. The bold and cursive values are close to 0.4.

| EPDS question | Depressed mood | Anxiety | Anhedonia |
| --- | --- | --- | --- |
| (1) I have been able to laugh and see the funny side of things. | -0.02 | 0.01 | **0.94** |
| (2) I have looked forward with enjoyment to things. | 0.16 | 0.01 | **0.63** |
| (3) I have blamed myself unnecessarily when things went wrong. | ***0.37*** | ***0.38*** | -0.03 |
| (4) I have been anxious or worried for no good reason. | -0.06 | **0.85** | 0.04 |
| (5) I have felt scared or panicky for no good reason. | 0.20 | **0.60** | -0.03 |
| (6) Things have been getting to me. | ***0.38*** | 0.17 | 0.16 |
| (7) I have been so unhappy that I have had difficulty sleeping. | **0.61** | 0.11 | 0.05 |
| (8) I have felt sad or miserable. | **0.66** | 0.02 | 0.21 |
| (9) I have been so unhappy that I have been crying. | **0.81** | -0.04 | -0.02 |
| (10) The thought of harming myself has occurred to me. | **0.44** | 0.07 | -0.04 |

**Table S6. Results of logistic regression models from each separate timepoint in BASIC and Mom2B.** The results are presented as odds ratios and 95% Confidence intervals retrieved from the separate logistic regression models implemented at each separate timepoint for both BASIC and Mom2B.

|  | OR | 95% CI | OR | 95% CI |
| --- | --- | --- | --- | --- |
| **BASIC cohort** | | | | |
|  | **PMDD** | | **PMS** | |
| *Pregnancy* |  |  |  |  |
| Week 17 | 2.62 | 1.96, 3.49 | 1.65 | 1.37, 2.00 |
| Week 32 | 3.05 | 2.26, 4.10 | 1.63 | 1.34, 1.98 |
| *Postpartum* |  |  |  |  |
| Week 6 | 2.76 | 2.03, 3.72 | 1.34 | 1.10, 1.64 |
| Month 6 | 2.31 | 1.66, 3.20 | 1.56 | 1.26, 1.93 |
| **Mom2B cohort** | | | | |
|  | **Severe PMS** | | **PMS** | |
| *Pregnancy* |  |  |  |  |
| Week 12-24 | - | - | - | - |
| Week 24-34 | 1.53 | 1.04, 2.23 | - | - |
| Week 36-42 | - | - | - | - |
| *Postpartum* |  |  |  |  |
| Week 1-4 | - | - | 1.37 | 1.04, 1.79 |
| Week 6-13 | - | - | 1.68 | 1.25, 2.29 |
| Week 14-23 | - | - | 1.43 | 1.03, 2.01 |

**Table S7. Number of individuals and proportions in each PND severity category in BASIC.** For each timepoint and severity category, the total number of individuals are presented, along with the number and proportion of the total among those reporting no premenstrual symptomatology, PMS and PMDD.

| **Pregnancy week 17** | **None N=3660** | | | **Mild to moderate**  **N=688** | | | **Moderate to severe**  **N=182** | | | **Very severe**  **N=26** | | |
| --- | --- | --- | --- | --- | --- | --- | --- | --- | --- | --- | --- | --- |
|  | No PMS | PMS | PMDD | No PMS | PMS | PMDD | No PMS | PMS | PMDD | No PMS | PMS | PMDD |
| N (%) | 2403  (66%) | 1071  (29%) | 166  (4.5%) | 388  (56%) | 210  (31%) | 87  (13%) | 81  (45%) | 65  (36%) | 35  (19%) | 14  (54%) | 8  (31%) | 4  (15%) |
| **Pregnancy week 32** | **None N=3552** | | | **Mild to moderate**  **N=728** | | | **Moderate to severe**  **N=185** | | | **Very severe**  **N=21** | | |
|  | No PMS | PMS | PMDD | No PMS | PMS | PMDD | No PMS | PMS | PMDD | No PMS | PMS | PMDD |
| N (%) | 2281  (64%) | 988  (28%) | 141  (4.0%) | 360  (49%) | 236  (32%) | 91  (13%) | 82  (44%) | 51  (28%) | 36  (19%) | 10  (48%) | 5  (24%) | 6  (29%) |
| **Postpartum week 6** | **None N=3469** | | | **Mild to moderate**  **N=709** | | | **Moderate to severe**  **N=189** | | | **Very severe**  **N=34** | | |
|  | No PMS | PMS | PMDD | No PMS | PMS | PMDD | No PMS | PMS | PMDD | No PMS | PMS | PMDD |
| N (%) | 2071  (60%) | 931  (27%) | 133  (3.8%) | 348  (49%) | 207  (29%) | 74  (10%) | 89  (47%) | 43  (23%) | 39  (21%) | 20  (59%) | 6  (18%) | 4  (12%) |
| **Postpartum month 6** | **None N=3232** | | | **Mild to moderate**  **N=579** | | | **Moderate to severe**  **N=177** | | | **Very severe**  **N=21** | | |
|  | No PMS | PMS | PMDD | No PMS | PMS | PMDD | No PMS | PMS | PMDD | No PMS | PMS | PMDD |
| N (%) | 1986  (61%) | 871  (27%) | 131  (4.1%) | 291  (50%) | 172  (30%) | 70  (12%) | 80  (45%) | 60  (34%) | 19  (16%) | 15  (71%) | 1  (4.8%) | 3  (14%) |

**Table S8.** **Chi-square results of comparisons of severity categories for BASIC.** The total chi-square statistic is presented, along with the chi-square contributions for each severity category and those reporting no premenstrual symptomatology, PMS and PMDD at each of the four timepoints, and the corresponding p-value.

|  | χ2 | | | | | | | | | | | | | df | p |
| --- | --- | --- | --- | --- | --- | --- | --- | --- | --- | --- | --- | --- | --- | --- | --- |
|  |  | **None** | | | **Mild-moderate** | | | **Moderate-severe** | | | **Very severe** | | |  |  |
|  | Total | No PMS | PMS | PMDD | No PMS | PMS | PMDD | No PMS | PMS | PMDD | No PMS | PMS | PMDD |  |  |
| *Pregnancy* |  |  |  |  |  |  |  |  |  |  |  |  |  |  |  |
| Week 17 | 133.2 | 3.1 | 0.25 | 20 | 5.3 | 0.1 | 42 | 10 | 2.2 | 47 | 0.4 | 0.007 | 3.2 | 6 | 2.2*10^-16^ |
| Week 32 | 184.6 | 5.3 | 0.9 | 27 | 14 | 4.6 | 51 | 6.1 | 0.006 | 59 | 0.9 | 0.3 | 16 | 6 | 2.2*10^-16^ |
| *Postpartum* |  |  |  |  |  |  |  |  |  |  |  |  |  |  |  |
| Week 6 | 144.7 | 2.6 | 0.06 | 21 | 7.0 | 1.9 | 30 | 3.7 | 1.3 | 74 | 0.04 | 1.0 | 2.4 | 6 | 2.2*10^-16^ |
| Month 6 | 116.9 | 3.0 | 0.4 | 17 | 7.3 | 1.1 | 40 | 7.3 | 1.9 | 32 | 0.7 | 3.8 | 2.7 | 6 | 2.2*10^-16^ |

**Table S9. Number of individuals and proportions in each PND severity category in Mom2B.** For each timepoint and severity category, the total number of individuals are presented, along with the number and proportion of the total among those reporting no premenstrual symptomatology, PMS and severe PMS.

| Pregnancy week 12-22 | None N=1813 | | | Mild to moderate  N=608 | | | Moderate to severe  N=210 | | | Very severe  N=37 | | |
| --- | --- | --- | --- | --- | --- | --- | --- | --- | --- | --- | --- | --- |
|  | No PMS | PMS | Severe  PMS | No PMS | PMS | Severe  PMS | No PMS | PMS | Severe  PMS | No PMS | PMS | Severe  PMS |
| N (%) | 646  (36%) | 919  (51%) | 123  (6.8%) | 147  (24%) | 357  (59%) | 56  (9.2%) | 43  (20%) | 116  (55%) | 32  (15%) | 7  (19%) | 20  (54%) | 5  (14%) |
| **Pregnancy week 24-34** | **None N=1981** | | | **Mild to moderate**  **N=674** | | | **Moderate to severe**  **N=250** | | | **Very severe**  **N=59** | | |
|  | No PMS | PMS | Severe  PMS | No PMS | PMS | Severe  PMS | No PMS | PMS | Severe  PMS | No PMS | PMS | Severe  PMS |
| N (%) | 718  (36%) | 984  (50%) | 141  (7.1%) | 189  (28%) | 375  (56%) | 55  (8.2%) | 58  (23%) | 126  (50%) | 41  (16%) | 20  (34%) | 28  (47%) | 6  (10%) |
| **Pregnancy week 36-42** | **None N=1615** | | | **Mild to moderate**  **N=499** | | | **Moderate to severe**  **N=143** | | | **Very severe**  **N=43** | | |
|  | No PMS | PMS | Severe  PMS | No PMS | PMS | Severe  PMS | No PMS | PMS | Severe  PMS | No PMS | PMS | Severe  PMS |
| N (%) | 596  (37%) | 812  (50%) | 123  (7.6%) | 123  (25%) | 289  (58%) | 48  (9.6%) | 36  (25%) | 80  (56%) | 14  (9.8%) | 14  (33%) | 23  (53%) | 3  (7.0%) |
| **Postpartum week 1-4** | **None N=1355** | | | **Mild to moderate**  **N=487** | | | **Moderate to severe**  **N=184** | | | **Very severe**  **N=35** | | |
|  | No PMS | PMS | Severe  PMS | No PMS | PMS | Severe  PMS | No PMS | PMS | Severe  PMS | No PMS | PMS | Severe  PMS |
| N (%) | 516  (38%) | 682  (50%) | 98  (7.2%) | 139  (29%) | 289  (59%) | 42  (8.6%) | 42  (23%) | 106  (58%) | 21  (11%) | 6  (17%) | 18  (51%) | 6  (17%) |
| **Postpartum week 6-13** | **None N=1411** | | | **Mild to moderate**  **N=366** | | | **Moderate to severe**  **N=133** | | | **Very severe**  **N=25** | | |
|  | No PMS | PMS | Severe  PMS | No PMS | PMS | Severe  PMS | No PMS | PMS | Severe  PMS | No PMS | PMS | Severe  PMS |
| N (%) | 546  (39%) | 700  (50%) | 102  (7.2%) | 102  (28%) | 215  (59%) | 33  (9.0%) | 25  (19%) | 73  (55%) | 17  (12.8%) | 5  (20%) | 15  (60%) | 4  (16%) |
| **Postpartum week 14-23** | **None N=1111** | | | **Mild to moderate**  **N=282** | | | **Moderate to severe**  **N=92** | | | **Very severe**  **N=21** | | |
|  | No PMS | PMS | Severe  PMS | No PMS | PMS | Severe  PMS | No PMS | PMS | Severe  PMS | No PMS | PMS | Severe  PMS |
| N (%) | 411  (37%) | 581  (52%) | 72  (6.5%) | 88  (31%) | 154  (55%) | 30  (11%) | 14  (15%) | 64  (70%) | 10  (11%) | 4  (19%) | 13  (62%) | 1  (4.8%) |

**Table S10. Chi-square results of comparisons of severity categories for Mom2B.** The total chi-square statistic is presented, along with the chi-square contributions for each severity category and those reporting no premenstrual symptomatology, PMS and severe PMS at each of the six timepoints, and the corresponding p-value.

|  | χ2 | | | | | | | | | | | | | df | p |
| --- | --- | --- | --- | --- | --- | --- | --- | --- | --- | --- | --- | --- | --- | --- | --- |
|  |  | **None** | | | **Mild-moderate** | | | **Moderate-severe** | | | **Very severe** | | |  |  |
|  | Total | No PMS | PMS | Severe PMS | No PMS | PMS | Severe PMS | No PMS | PMS | Severe PMS | No PMS | PMS | Severe PMS |  |  |
| *Pregnancy* |  |  |  |  |  |  |  |  |  |  |  |  |  |  |  |
| Week 12-22 | 55.5 | 8.5 | 2.2 | 4.1 | 10 | 4.3 | 1.0 | 7.5 | 0.4 | 14 | 1.4 | 0.2 | 1.7 | 6 | 3.63*10^-10^ |
| Week 24-34 | 46.3 | 4.7 | 1.1 | 3.1 | 5.0 | 3.2 | 0 | 6.5 | 0.03 | 22 | 0.02 | 0.1 | 0.3 | 6 | 2.62*10^-8^ |
| Week 36-42 | 27.2 | 4.8 | 2.0 | 0.8 | 10 | 4.2 | 1.6 | 2.3 | 0.8 | 0.6 | 0.004 | 0.02 | 0.1 | 6 | 1.30*10^-4^ |
| *Postpartum* |  |  |  |  |  |  |  |  |  |  |  |  |  |  |  |
| Week 1-4 | 34.5 | 5.9 | 2.2 | 1.3 | 5.1 | 2.8 | 0.1 | 5.6 | 1.5 | 3.1 | 2.1 | 0.1 | 4.7 | 6 | 5.41*10^-6^ |
| Week 6-13 | 35.0 | 4.7 | 1.8 | 1.4 | 5.7 | 3.0 | 0.4 | 7.2 | 1.7 | 5.4 | 1.7 | 0.3 | 1.9 | 6 | 4.42*10^-6^ |
| Week 14-23 | 26.1 | 2.3 | 0.6 | 1.6 | 0.9 | 0.005 | 3.5 | 9.8 | 4.2 | 1.4 | 0.9 | 0.8 | 0.1 | 6 | 2.14*10^-4^ |

**Table S11. Proportions of PND trajectories and results from multinomial logistic regression for BASIC.** For each PND trajectory and premenstrual symptomatology group, n:s are presented together with row (R) and column (C) percentages of the proportion by the total n of the row or column. For gestational PND, postpartum PND, and persistent PND, ORs with 95% CI is presented, derived from two separate multinomial logistic regression models, using PMS (yes/no) and PMDD (yes/no) as the independent variable, and a variable including the trajectories as the dependent variable, with the control trajectory as baseline. Results with ‘**’ indicate p≤0.01, those with ‘***’ indicate p≤0.001, and those with ‘****’ indicate p≤0.0001. The highlighted cells are the significant results.

|  | Controls  (n = 2363) | | Gestational PND  (n = 290) | | Postpartum PND  (n = 406) | | Persistent PND  (n = 67) | |
| --- | --- | --- | --- | --- | --- | --- | --- | --- |
|  | n  (R %; C%) | OR  [95% CI] | n  (R %; C%) | OR  (95% CI) | n  (R %;C%) | OR  (95% CI) | n  (R %; C%) | OR  (95% CI) |
| No PMS  (n = 2044) | 1617  (R:79%; C:68%) | - | 151  (R:7.4%; C:52%) | - | 234  (R:11%; C:58%) | - | 42  (R:2.1%; C:63%) | - |
| PMS  (n = 916) | 681  (R:74%; C:29%) | - | 97  (R:11%; C:33%) | 1.5  [1.2, 2.0]^**^ | 121  (R:13%; C:30%) | 1.2  [0.97, 1.6] | 17  (R:1.9%; C:25%) | 0.96  [0.54, 1.7] |
| PMDD  (n = 166) | 65  (R:39%; C:2.8%) | - | 42  (R:25%; C:15%) | 6.9  [4.5, 10]^****^ | 51  (R:31%; C:13%) | 5.4  [3.7, 8.0]^****^ | 8  (R:4.8%; C:12%) | 4.7  [2.1, 10]^***^ |

These trajectories are only based on the participants fitting into the specified groups of symptoms onset and persistence.

**Table S12. Proportions of PND trajectories and results from multinomial logistic regression for Mom2B.** For each PND trajectory and premenstrual symptomatology group, n:s are presented together with row (R) and column (C) percentages of the proportion by the total n of the row or column. For gestational PND, postpartum PND, and persistent PND, ORs with 95% CI is presented, derived from two separate multinomial logistic regression models, using PMS (yes/no) and PMDD (yes/no) as the independent variable, and a variable including the trajectories as the dependent variable, with the control trajectory as baseline. Results with ‘*’ indicate p≤0.05, and those with ‘**’ indicate p≤0.01. The highlighted cells are the significant results.

|  | Controls  (n = 311) | | Gestational PND  (n = 137) | | Postpartum PND  (n = 116) | | Persistent PND  (n = 17) | |
| --- | --- | --- | --- | --- | --- | --- | --- | --- |
|  | n  (R %; C%) | OR  [95% CI] | n  (R %; C%) | OR  (95% CI) | n  (R %;C%) | OR  (95% CI) | n  (R %; C%) | OR  (95% CI) |
| No PMS  (n = 200) | 119  (R:60%; C:38%) | - | 43  (R:22%; C:31%) | - | 35  (R:18%; C:30%) | - | 3  (R:1.5%; C:18%) | - |
| PMS  (n = 329) | 173  (R:53%; C:56%) | - | 80  (R:24%; C:58%) | 1.3  [0.83, 2.0] | 66  (R:20%; C:57%) | 1.3  [0.81, 2.1] | 10  (R:3.0%; C:59%) | 2.3  [0.62, 8.5] |
| Severe PMS  (n = 52) | 19  (R:37%; C:6.1%) | - | 14  (R:27%; C:10%) | 2.0  [0.94, 4.4] | 15  (R:29%; C:13%) | 2.7  [1.2, 5.8]^*^ | 4  (R:7.7%;  C:24%) | 8.4  [1.7, 40]^**^ |

These trajectories are only based on the participants fitting into the specified groups of symptoms onset and persistence.

**Table S13.** **Number of individuals and proportions in each dimensional phenotype of PND in BASIC.** For each timepoint and dimensional phenotype, the total number of individuals are presented, along with the number and proportion of the total among those reporting no premenstrual symptomatology, PMS and PMDD.

| Pregnancy week 17 | No PND N=1474 | | | Mild depression  N=1370 | | | Depression with anhedonia  N=533 | | | Moderate depression with anxiety  N=628 | | | Moderate depression with anxiety and anhedonia  N=370 | | | Severe depression with anxiety and anhedonia  N=181 | | |
| --- | --- | --- | --- | --- | --- | --- | --- | --- | --- | --- | --- | --- | --- | --- | --- | --- | --- | --- |
|  | No PMS | PMS | PMDD | No PMS | PMS | PMDD | No PMS | PMS | PMDD | No PMS | PMS | PMDD | No PMS | PMS | PMDD | No PMS | PMS | PMDD |
| N (%) | 1041 (71%) | 396  (27%) | 30 (2.0%) | 876 (64%) | 412 (30%) | 73 (5.3%) | 315  (59%) | 171  (32%) | 42  (7.9%) | 369 (59%) | 200  (32%) | 59  (9.4%) | 195  (53%) | 113  (31%) | 60  (16%) | 90  (50%) | 62  (34%) | 28  (15%) |
| **Pregnancy week 32** | **No PND N=1268** | | | **Mild depression**  **N=1439** | | | **Depression with anhedonia**  **N=643** | | | **Moderate depression with anxiety**  **N=563** | | | **Moderate depression with anxiety and anhedonia**  **N=375** | | | **Severe depression with anxiety and anhedonia**  **N=198** | | |
|  | No PMS | PMS | PMDD | No PMS | PMS | PMDD | No PMS | PMS | PMDD | No PMS | PMS | PMDD | No PMS | PMS | PMDD | No PMS | PMS | PMDD |
| N (%) | 900 (71%) | 303  (24%) | 17  (1.3%) | 899  (62%) | 408  (28%) | 71  (5.0%) | 360  (56%) | 212  (33%) | 48  (7.5%) | 309  (55%) | 178  (32%) | 47  (8.3%) | 176  (47%) | 122  (33%) | 51  (14%) | 89  (45%) | 57  (29%) | 39  (20%) |
| **Postpartum week 6** | **No PND N=1136** | | | **Mild depression**  **N=1520** | | | **Depression with anhedonia**  **N=621** | | | **Moderate depression with anxiety**  **N=545** | | | **Moderate depression with anxiety and anhedonia**  **N=350** | | | **Severe depression with anxiety and anhedonia N=229** | | |
|  | No PMS | PMS | PMDD | No PMS | PMS | PMDD | No PMS | PMS | PMDD | No PMS | PMS | PMDD | No PMS | PMS | PMDD | No PMS | PMS | PMDD |
| N (%) | 722  (48%) | 291  (19%) | 19  (1.3%) | 903  (59%) | 393  (26%) | 72  (4.7%) | 328  (53%) | 186  (30%) | 39  (6.3%) | 282  (52%) | 169  (31%) | 40  (7.3%) | 170  (49%) | 94  (27%) | 45  (13%) | 123  (54%) | 54  (24%) | 35  (15%) |
| **Postpartum month 6** | **No PND N=1246** | | | **Mild depression**  **N=1289** | | | **Depression with anhedonia**  **N=545** | | | **Moderate depression with anxiety**  **N=470** | | | **Moderate depression with anxiety and anhedonia**  **N=270** | | | **Severe depression with anxiety and anhedonia**  **N=189** | | |
|  | No PMS | PMS | PMDD | No PMS | PMS | PMDD | No PMS | PMS | PMDD | No PMS | PMS | PMDD | No PMS | PMS | PMDD | No PMS | PMS | PMDD |
| N (%) | 802 (62%) | 321  (25%) | 29  (2.2%) | 796  (62%) | 349  (27%) | 58  (4.5%) | 308  (57%) | 155  (28%) | 38  (7.0%) | 255  (54%) | 136  (29%) | 34  (7.3%) | 123  (46%) | 85  (31%) | 40  (21%) | 88  (47%) | 58  (31%) | 34  (18%) |

**Table S14. Number of individuals and proportions in each dimensional phenotype of PND in Mom2B.** For each timepoint and dimensional phenotype, the total number of individuals are presented, along with the number and proportion of the total among those reporting no premenstrual symptomatology, PMS and severe PMS.

| Pregnancy week 12-22 | No PND  N=757 | | | Mild depression  N=504 | | | Mild anxiety  N=514 | | | Moderate depression  with anxiety  N=457 | | | Moderate depression with anxiety and anhedonia  N=234 | | | Severe depression with anxiety and anhedonia  N=202 | | | |
| --- | --- | --- | --- | --- | --- | --- | --- | --- | --- | --- | --- | --- | --- | --- | --- | --- | --- | --- | --- |
|  | No PMS | PMS | Severe  PMS | No PMS | PMS | Severe  PMS | No PMS | PMS | Severe  PMS | No PMS | PMS | Severe  PMS | No PMS | PMS | Severe  PMS | No PMS | PMS | Severe  PMS |  |
| **N (%)** | 328  (43%) | 337  (45%) | 42  (5.5%) | 142  (28%) | 295  (59%) | 42  (8.3%) | 160  (31%) | 278  (54%) | 34  (6.6%) | 109  (24%) | 259  (57%) | 43  (9.4%) | 67  (29%) | 130  (56%) | 23  (9.8%) | 37  (18%) | 113  (56%) | 32  (16%) |  |
| **Pregnancy week 24-34** | **No PND**  **N=913** | | | **Mild depression**  **N=520** | | | **Mild anxiety**  **N=524** | | | **Moderate depression with anxiety**  **N=539** | | | **Moderate depression with anxiety and anhedonia**  **N=208** | | | **Severe depression with anxiety and anhedonia**  **N=260** | | | |
|  | No PMS | PMS | Severe  PMS | No PMS | PMS | Severe  PMS | No PMS | PMS | Severe  PMS | No PMS | PMS | Severe  PMS | No PMS | PMS | Severe  PMS | No PMS | PMS | Severe  PMS |  |
| **N (%)** | 394  (43%) | 399  (44%) | 59  (6.5%) | 174  (33%) | 270  (52%) | 47  (9.0%) | 136  (26%) | 310  (59%) | 34  (6.5%) | 150  (28%) | 301  (56%) | 49  (9.1%) | 67  (32%) | 98  (47%) | 18  (8.7%) | 64  (25%) | 135  (52%) | 36  (14%) |  |
| **Pregnancy week 36-42** | **No PND**  **N=729** | | | **Mild depression**  **N=475** | | | **Mild anxiety**  **N=383** | | | **Moderate depression with anxiety**  **N=402** | | | **Moderate depression with anxiety and anhedonia**  **N=158** | | | **Severe depression with anxiety and anhedonia**  **N=153** | | | |
|  | No PMS | PMS | Severe  PMS | No PMS | PMS | Severe  PMS | No PMS | PMS | Severe  PMS | No PMS | PMS | Severe  PMS | No PMS | PMS | Severe  PMS | No PMS | PMS | Severe  PMS |  |
| **N (%)** | 314  (43%) | 331  (45%) | 50  (6.9%) | 150  (32%) | 257  (54%) | 37  (7.8%) | 117  (31%) | 216  (56%) | 32  (8.4%) | 113  (28%) | 221  (55%) | 40  (10%) | 36  (23%) | 96  (61%) | 12  (7.6%) | 39  (25%) | 83  (54%) | 17  (11%) |  |
| **Postpartum week 1-4** | **No PND**  **N=557** | | | **Mild depression**  **N=374** | | | **Mild anxiety**  **N=386** | | | **Moderate depression with anxiety**  **N=449** | | | **Moderate depression with anxiety and anhedonia**  **N=120** | | | **Severe depression with anxiety and anhedonia**  **N=175** | | | |
|  | No PMS | PMS | Severe  PMS | No PMS | PMS | Severe  PMS | No PMS | PMS | Severe  PMS | No PMS | PMS | Severe  PMS | No PMS | PMS | Severe  PMS | No PMS | PMS | Severe  PMS |  |
| **N (%)** | 248  (45%) | 258  (46%) | 29  (5.2%) | 137  (37%) | 199  (53%) | 26  (7.0%) | 124  (32%) | 205  (53%) | 38  (9.8%) | 119  (27%) | 267  (59%) | 38  (8.5%) | 37  (31%) | 67  (56%) | 15  (13%) | 38  (22%) | 99  (57%) | 21  (12%) |  |
| **Postpartum week 6-13** | **No PND**  **N=668** | | | **Mild depression**  **N=416** | | | **Mild anxiety**  **N=294** | | | **Moderate depression with anxiety**  **N=304** | | | **Moderate depression with anxiety and anhedonia**  **N=129** | | | **Severe depression with anxiety and anhedonia**  **N=124** | | | |
|  | No PMS | PMS | Severe  PMS | No PMS | PMS | Severe  PMS | No PMS | PMS | Severe  PMS | No PMS | PMS | Severe  PMS | No PMS | PMS | Severe  PMS | No PMS | PMS | Severe  PMS |  |
| **N (%)** | 307  (46%) | 290  (43%) | 44  (6.6%) | 141  (34%) | 229  (55%) | 29  (7.0%) | 86  (29%) | 167  (57%) | 25  (8.5%) | 78  (26%) | 182  (60%) | 30  (9.9%) | 43  (33%) | 65  (50%) | 12  (9.3%) | 23  (19%) | 70  (56%) | 16  (13%) |  |
| **Postpartum week 14-23** | **No PND**  **N=571** | | | **Mild depression**  **N=308** | | | **Mild anxiety**  **N=210** | | | **Moderate depression with anxiety**  **N=240** | | | **Moderate depression with anxiety and anhedonia**  **N=90** | | | **Severe depression with anxiety and anhedonia**  **N=87** | | | |
|  | No PMS | PMS | Severe  PMS | No PMS | PMS | Severe  PMS | No PMS | PMS | Severe  PMS | No PMS | PMS | Severe  PMS | No PMS | PMS | Severe  PMS | No PMS | PMS | Severe  PMS |  |
| **N (%)** | 239  (42%) | 278  (49%) | 28  (4.9%) | 108  (35%) | 165  (54%) | 23  (7.5%) | 53  (25%) | 124  (59%) | 23  (11%) | 72  (30%) | 136  (57%) | 24  (10%) | 33  (37%) | 47  (52%) | 8  (8.9%) | 12  (14%) | 62  (71%) | 7  (8.0%) |  |

**Table S15. Chi-square results of comparisons of dimensional phenotypes of PND for BASIC.** The total chi-square statistic is presented, along with the chi-square contributions for each dimensional phenotype and those reporting no premenstrual symptomatology, PMS and PMDD at each of the four timepoints, and the corresponding p-value.

|  | | Pregnancy | | Postpartum | |
| --- | --- | --- | --- | --- | --- |
|  |  | ***Week 17*** | ***Week 32*** | ***Week 6*** | ***Month 6*** |
| **χ2** | **Total** | 171.0 | 218.7 | 132.4 | 144.5 |
|  | **No PND** | | | | |
|  | No PMS | 12 | 19 | 6.2 | 5.8 |
|  | PMS | 4.1 | 10 | 1.0 | 1.4 |
|  | PMDD | 44 | 47 | 33 | 26 |
|  | **Mild depression** | | | | |
|  | No PMS | 0.1 | 0.4 | 1.1 | 0.9 |
|  | PMS | 0.1 | 0.03 | 0.7 | 0.2 |
|  | PMDD | 2.5 | 3.0 | 2.4 | 4.1 |
|  | **Depression with anhedonia** | | | | |
|  | No PMS | 1.3 | 3.1 | 1.7 | 0.5 |
|  | PMS | 1.1 | 3.9 | 2.5 | 0.2 |
|  | PMDD | 1.9 | 1.8 | 0.5 | 1.4 |
|  | **Moderate depression with anxiety** | | | | |
|  | No PMS | 2.4 | 2.9 | 3.1 | 1.0 |
|  | PMS | 0.8 | 2.2 | 3.3 | 0.7 |
|  | PMDD | 8.5 | 4.9 | 2.6 | 2.0 |
|  | **Moderate depression with anxiety and anhedonia** | | | | |
|  | No PMS | 6.6 | 9.7 | 3.7 | 8.0 |
|  | PMS | 0.1 | 3.0 | 0.02 | 1.7 |
|  | PMDD | 56 | 37 | 33 | 38 |
|  | **Severe depression with anxiety and anhedonia** | | | | |
|  | No PMS | 5.3 | 7.1 | 1.1 | 6.4 |
|  | PMS | 1.3 | 0.1 | 1.4 | 0.4 |
|  | PMDD | 23 | 62 | 35 | 46 |
| **Df** | | 10 | 10 | 10 | 10 |
| **p** | | 2.2*10^-16^ | 2.2*10^-16^ | 2.2*10^-16^ | 2.2*10^-16^ |

**Table S16. Chi-square results of comparisons of dimensional phenotypes of PND for Mom2B.** The total chi-square statistic is presented, along with the chi-square contributions for each dimensional phenotype and those reporting no premenstrual symptomatology, PMS and severe PMS at each of the six timepoints, and the corresponding p-value.

|  | | Pregnancy | | | Postpartum | | |
| --- | --- | --- | --- | --- | --- | --- | --- |
|  |  | ***Week 12-22*** | ***Week 24-34*** | ***Week 36-42*** | ***Week 1-4*** | ***Week 6-13*** | ***Week 14-23*** |
| **χ2** | **Total** | 94.5 | 81.2 | 48.6 | 57.2 | 66.2 | 46.9 |
|  | **No PND** | | | | | | |
|  | No PMS | 31 | 25 | 18 | 17 | 21 | 9.7 |
|  | PMS | 11 | 11 | 8.2 | 5.4 | 10 | 2.7 |
|  | Severe PMS | 6.3 | 3.6 | 1.8 | 6.0 | 2.0 | 5.1 |
|  | **Mild depression** | | | | | | |
|  | No PMS | 2.8 | 0.03 | 0.4 | 0.4 | 0.3 | 0.03 |
|  | PMS | 1.7 | 0.004 | 0.4 | 0.04 | 0.6 | 0.02 |
|  | Severe PMS | 0 | 0.3 | 0.1 | 0.7 | 0.7 | 0.002 |
|  | **Mild anxiety** | | | | | | |
|  | No PMS | 0.007 | 7.7 | 1.3 | 0.4 | 2.7 | 4.9 |
|  | PMS | 0.3 | 7.7 | 0.8 | 0.001 | 1.5 | 1.2 |
|  | Severe PMS | 1.3 | 1.7 | 0.002 | 1.5 | 0.08 | 3.4 |
|  | **Moderate depression with anxiety** | | | | | | |
|  | No PMS | 6.9 | 4.9 | 3.0 | 7.0 | 7.9 | 1.5 |
|  | PMS | 2.5 | 2.3 | 0.8 | 4.0 | 3.5 | 0.2 |
|  | Severe PMS | 1.4 | 0.5 | 1.7 | 0.1 | 1.2 | 1.9 |
|  | **Moderate depression with anxiety and anhedonia** | | | | | | |
|  | No PMS | 0.9 | 0.02 | 4.5 | 0.7 | 0.04 | 0.07 |
|  | PMS | 0.1 | 0.09 | 3.1 | 0.007 | 0.004 | 0.1 |
|  | Severe PMS | 0.7 | 0.2 | 0.02 | 2.4 | 0.3 | 0.2 |
|  | **Severe depression with anxiety and anhedonia** | | | | | | |
|  | No PMS | 10 | 5.0 | 2.2 | 6.0 | 7.4 | 10 |
|  | PMS | 0.8 | 0.2 | 0.4 | 1.4 | 1.8 | 5.9 |
|  | Severe PMS | 16 | 11 | 2.0 | 4.3 | 4.9 | 0.1 |
| **Df** | | 10 | 10 | 10 | 10 | 10 | 10 |
| **p** | | 6.96*10^-16^ | 2.91*10^-13^ | 4.74*10^-7^ | 1.25*10^-8^ | 2.43*10^-10^ | 9.70*10^-7^ |

**Table S17. PND phenotypes in relation to PND severity over time for BASIC.** The number of those in each severity category and in each dimensional phenotype of PND, and the proportion based on the total number in each dimensional phenotype, are presented for each of the four timepoints in BASIC.

| **BASIC cohort: Week 17 of pregnancy** | | | | | | |
| --- | --- | --- | --- | --- | --- | --- |
|  | **No PND**,  N = 1474 | **Mild depression**,  N = 1370 | **Depression with anhedonia**,  N = 533 | **Moderate depression with anxiety**,  N = 628 | **Moderate depression with anxiety and anhedonia**,  N = 370 | **Severe depression with anxiety and anhedonia**,  N = 181 |
| **Severity*** |  |  |  |  |  |  |
| None | 1474 (100%) | 1370 (100%) | 330 (62%) | 483 (77%) | 3 (0.8%) | 0 (0%) |
| Mild to moderate | 0 (0%) | 0 (0%) | 203 (38%) | 145 (23%) | 311 (84%) | 29 (16%) |
| Moderate to severe | 0 (0%) | 0 (0%) | 0 (0%) | 0 (0%) | 56 (15%) | 126 (70%) |
| Very severe | 0 (0%) | 0 (0%) | 0 (0%) | 0 (0%) | 0 (0%) | 26 (14%) |

| **BASIC cohort: Week 32 of pregnancy** | | | | | | |
| --- | --- | --- | --- | --- | --- | --- |
|  | **No PND**,  N = 1268 | **Mild depression**,  N = 1439 | **Depression with anhedonia**,  N = 643 | **Moderate depression with anxiety**,  N = 563 | **Moderate depression with anxiety and anhedonia**,  N = 375 | **Severe depression with anxiety and anhedonia**,  N = 198 |
| **Severity*** |  |  |  |  |  |  |
| None | 1268 (100%) | 1439 (100%) | 410 (64%) | 429 (76%) | 6 (1.6%) | 0 (0%) |
| Mild to moderate | 0 (0%) | 0 (0%) | 233 (36%) | 134 (24%) | 317 (85%) | 44 (22%) |
| Moderate to severe | 0 (0%) | 0 (0%) | 0 (0%) | 0 (0%) | 52 (14%) | 133 (67%) |
| Very severe | 0 (0%) | 0 (0%) | 0 (0%) | 0 (0%) | 0 (0%) | 21 (11%) |

| **BASIC cohort: Postpartum week 6** | | | | | | |
| --- | --- | --- | --- | --- | --- | --- |
|  | **No PND**,  N = 1136 | **Mild depression**,  N = 1520 | **Depression with anhedonia**,  N = 621 | **Moderate depression with anxiety**,  N = 545 | **Moderate depression with anxiety and anhedonia**,  N = 350 | **Severe depression with anxiety and anhedonia**,  N = 229 |
| **Severity*** |  |  |  |  |  |  |
| None | 1136 (100%) | 1520 (100%) | 382 (62%) | 431 (79%) | 0 (0%) | 0 (0%) |
| Mild to moderate | 0 (0%) | 0 (0%) | 239 (38%) | 114 (21%) | 298 (85%) | 58 (25%) |
| Moderate to severe | 0 (0%) | 0 (0%) | 0 (0%) | 0 (0%) | 52 (15%) | 137 (60%) |
| Very severe | 0 (0%) | 0 (0%) | 0 (0%) | 0 (0%) | 0 (0%) | 34 (15%) |

| **BASIC cohort: Postpartum month 6** | | | | | | |
| --- | --- | --- | --- | --- | --- | --- |
|  | **No PND**,  N = 1246 | **Mild depression**,  N = 1289 | **Depression with anhedonia**,  N = 545 | **Moderate depression with anxiety**,  N = 470 | **Moderate depression with anxiety and anhedonia**,  N = 270 | **Severe depression with anxiety and anhedonia**,  N = 189 |
| **Severity*** |  |  |  |  |  |  |
| None | 1246 (100%) | 1289 (100%) | 336 (62%) | 360 (77%) | 1 (0.4%) | 0 (0%) |
| Mild to moderate | 0 (0%) | 0 (0%) | 209 (38%) | 110 (23 %) | 217 (80%) | 43 (23 %) |
| Moderate to severe | 0 (0%) | 0 (0%) | 0 (0%) | 0 (0%) | 52 (19%) | 125 (66%) |
| Very severe | 0 (0%) | 0 (0%) | 0 (0%) | 0 (0%) | 0 (0%) | 21 (11%) |

* Percentages based on clusters

**Table S18. PND phenotypes in relation to PND severity over time for Mom2B.** The number of those in each severity category and in each dimensional phenotype of PND, and the proportion based on the total number in each dimensional phenotype, are presented for each of the six timepoints.

| **Mom2B cohort: Week 12-22 of pregnancy** | | | | | | |
| --- | --- | --- | --- | --- | --- | --- |
|  | **No PND**,  N = 757 | **Mild depression**,  N = 504 | **Mild anxiety,**  N = 514 | **Moderate depression with anxiety**,  N = 457 | **Moderate depression with anxiety and anhedonia**,  N = 234 | **Severe depression with anxiety and anhedonia**,  N = 202 |
| **Severity*** |  |  |  |  |  |  |
| None | 757 (100%) | 484 (96%) | 477 (93%) | 66 (14%) | 29 (12%) | 0 (0%) |
| Mild to moderate | 0 (0%) | 20 (4.0%) | 37 (7.2%) | 365 (80%) | 185 (79%) | 1 (0.5 %) |
| Moderate to severe | 0 (0%) | 0 (0%) | 0 (0%) | 26 (5.7%) | 20 (8.5%) | 164 (81%) |
| Very severe | 0 (0%) | 0 (0%) | 0 (0%) | 0 (0%) | 0 (0%) | 37 (18%) |

| **Mom2B cohort: Week 24-34 of pregnancy** | | | | | | |
| --- | --- | --- | --- | --- | --- | --- |
|  | **No PND**,  N = 913 | **Mild depression**,  N = 520 | **Mild anxiety,**  N = 524 | **Moderate depression with anxiety**,  N = 539 | **Moderate depression with anxiety and anhedonia**,  N = 208 | **Severe depression with anxiety and anhedonia**,  N = 260 |
| **Severity*** |  |  |  |  |  |  |
| None | 913 (100%) | 492 (95%) | 488 (93%) | 76 (14%) | 12 (5.8%) | 0 (0%) |
| Mild to moderate | 0 (0%) | 28 (5.4%) | 36 (6.9%) | 433 (80%) | 173 (83%) | 4 (1.5 %) |
| Moderate to severe | 0 (0%) | 0 (0%) | 0 (0%) | 30 (5.6%) | 23 (11%) | 197 (76%) |
| Very severe | 0 (0%) | 0 (0%) | 0 (0%) | 0 (0%) | 0 (0%) | 59 (23%) |

| **Mom2B cohort: Week 36-42 of pregnancy** | | | | | | |
| --- | --- | --- | --- | --- | --- | --- |
|  | **No PND**,  N = 729 | **Mild depression**,  N = 475 | **Mild anxiety,**  N = 383 | **Moderate depression with anxiety**,  N = 402 | **Moderate depression with anxiety and anhedonia**,  N = 158 | **Severe depression with anxiety and anhedonia**,  N = 153 |
| **Severity*** |  |  |  |  |  |  |
| None | 729 (100%) | 463 (97%) | 353 (92%) | 58 (14%) | 12 (7.6%) | 0 (0%) |
| Mild to moderate | 0 (0%) | 12 (2.5%) | 30 (7.8%) | 323 (80%) | 132 (84%) | 2 (1.3 %) |
| Moderate to severe | 0 (0%) | 0 (0%) | 0 (0%) | 21 (5.2%) | 14 (8.9%) | 108 (71%) |
| Very severe | 0 (0%) | 0 (0%) | 0 (0%) | 0 (0%) | 0 (0%) | 43 (28%) |

| **Mom2B cohort: Postpartum week 1-4** | | | | | | |
| --- | --- | --- | --- | --- | --- | --- |
|  | **No PND**,  N = 557 | **Mild depression**,  N = 374 | **Mild anxiety,**  N = 386 | **Moderate depression with anxiety**,  N = 449 | **Moderate depression with anxiety and anhedonia**,  N = 120 | **Severe depression with anxiety and anhedonia**,  N = 175 |
| **Severity*** |  |  |  |  |  |  |
| None | 557 (100%) | 358 (96%) | 360 (93%) | 72 (16%) | 8 (6.7%) | 0 (0%) |
| Mild to moderate | 0 (0%) | 16 (4.3%) | 26 (6.7%) | 351 (78%) | 91 (76%) | 3 (1.7 %) |
| Moderate to severe | 0 (0%) | 0 (0%) | 0 (0%) | 26 (5.8%) | 21 (18%) | 137 (78%) |
| Very severe | 0 (0%) | 0 (0%) | 0 (0%) | 0 (0%) | 0 (0%) | 35 (20%) |

| **Mom2B cohort: Postpartum week 6-13** | | | | | | |
| --- | --- | --- | --- | --- | --- | --- |
|  | **No PND**,  N = 668 | **Mild depression**,  N = 416 | **Mild anxiety,**  N = 294 | **Moderate depression with anxiety**,  N = 304 | **Moderate depression with anxiety and anhedonia**,  N = 129 | **Severe depression with anxiety and anhedonia**,  N = 124 |
| **Severity*** |  |  |  |  |  |  |
| None | 668 (100%) | 401 (96%) | 282 (96%) | 50 (16%) | 10 (7.8%) | 0 (0%) |
| Mild to moderate | 0 (0%) | 15 (3.6%) | 12 (4.1%) | 238 (78%) | 95 (74%) | 6 (4.8 %) |
| Moderate to severe | 0 (0%) | 0 (0%) | 0 (0%) | 16 (5.3%) | 24 (19%) | 93 (75%) |
| Very severe | 0 (0%) | 0 (0%) | 0 (0%) | 0 (0%) | 0 (0%) | 25 (20%) |

| **Mom2B cohort: Postpartum week 14-23** | | | | | | |
| --- | --- | --- | --- | --- | --- | --- |
|  | **No PND**,  N = 571 | **Mild depression**,  N = 308 | **Mild anxiety,**  N = 210 | **Moderate depression with anxiety**,  N = 240 | **Moderate depression with anxiety and anhedonia**,  N = 90 | **Severe depression with anxiety and anhedonia**,  N = 87 |
| **Severity*** |  |  |  |  |  |  |
| None | 571 (100%) | 297 (96%) | 204 (97%) | 30 (13%) | 9 (10%) | 0 (0%) |
| Mild to moderate | 0 (0%) | 11 (3.6%) | 6 (2.9%) | 197 (82%) | 67 (74%) | 1 (1.1 %) |
| Moderate to severe | 0 (0%) | 0 (0%) | 0 (0%) | 13 (5.4%) | 14 (16%) | 65 (75%) |
| Very severe | 0 (0%) | 0 (0%) | 0 (0%) | 0 (0%) | 0 (0%) | 21 (24%) |

* Percentages based on clusters

# **References**

1. Axfors C, Bränn E, Henriksson HE, Hellgren C, Kunovac Kallak T, Fransson E, et al. Cohort profile: the Biology, Affect, Stress, Imaging and Cognition (BASIC) study on perinatal depression in a population-based Swedish cohort. BMJ Open. 2019;9(10):e031514.

2. A.P.A. Diagnostic and Statistical Manual of Mental Disorders, 5th edition (DSM-5): American Psychiatric Association; 2013.

3. Greenwald R, Rubin A. Assessment of Posttraumatic Symptoms in Children: Development and Preliminary Validation of Parent and Child Scales. Research on Social Work Practice. 1999;9(1):61-75.

4. Bilal AM, Fransson E, Bränn E, Eriksson A, Zhong M, Gidén K, et al. Predicting perinatal health outcomes using smartphone-based digital phenotyping and machine learning in a prospective Swedish cohort (Mom2B): study protocol. BMJ Open. 2022;12(4):e059033.

5. Cox JL, Holden JM, Sagovsky R. Detection of Postnatal Depression: Development of the 10-item Edinburgh Postnatal Depression Scale. The British Journal of Psychiatry. 1987;150(6):782-6.

6. World Health Organization. International statistical classification of diseases and related health problems (11th ed.). <https://icd.who.int/> 2019

7. Insel T, Cuthbert B, Garvey M, Heinssen R, Pine DS, Quinn K, et al. Research domain criteria (RDoC): toward a new classification framework for research on mental disorders. Am J Psychiatry. 2010;167(7):748-51.

8. Putnam KT, Wilcox M, Robertson-Blackmore E, Sharkey K, Bergink V, Munk-Olsen T, et al. Clinical phenotypes of perinatal depression and time of symptom onset: analysis of data from an international consortium. The Lancet Psychiatry. 2017;4(6):477-85.
